# A Mechanistic Review on Toxicity Effects of Methamphetamine

**DOI:** 10.7150/ijms.99159

**Published:** 2025-01-01

**Authors:** Fitri Fareez Ramli, Purwo Sri Rejeki, Nurul 'Izzah Ibrahim, Gulnar Abdullayeva, Shariff Halim

**Affiliations:** 1 Department of Pharmacology, Faculty of Medicine, Universiti Kebangsaan Malaysia, 56000 Kuala Lumpur, Malaysia.; 2 Department of Psychiatry, University of Oxford, Warneford Hospital, Oxford OX3 7JX, UK.; 3 Physiology Division, Department of Medical Physiology and Biochemistry, Faculty of Medicine, Universitas Airlangga, Surabaya 60132, Indonesia.; 4 Old Road Campus Research Building, Department of Oncology, University of Oxford, Oxford OX3 7DQ, UK.; 5 Institute of Molecular Biology & Biotechnologies, Ministry of Science and Education of the Republic of Azerbaijan, 11 Izzat Nabiyev Str., AZ1073, Baku, Azerbaijan.; 6 Oxford Vaccine Group, Department of Paediatrics, University of Oxford, OX3 7TY, UK.; 7 Faculty of Health Sciences, Universiti Teknologi MARA Cawangan Pulau Pinang, Kampus Bertam, 13200 Pulau Pinang, Malaysia.

**Keywords:** cardiotoxicity, hepatotoxicity, renal, meth, neurotoxicity

## Abstract

Persistent methamphetamine use causes many toxic effects in various organs, including the brain, heart, liver, kidney and eyes. The extent of its toxicity depends on numerous pharmacological factors, including route of administration, dose, genetic polymorphism related to drug metabolism and polysubstance abuse. Several molecular pathways have been proposed to activate oxidative stress, inflammation and apoptosis: B-cell lymphoma protein 2 (Bcl-2)-associated X (Bax)/Bcl2/caspase-3, nuclear factor erythroid 2-related factor (Nrf2)/heme oxygenase-1 (HO-1), protein kinase B (Akt)/mammalian target of rapamycin (mTOR)/p70S6K, trace amine-associated receptor 1 (TAAR1)/cAMP/lysyl oxidase, Sigmar1/ cAMP response element-binding protein (CREB)/mitochondrial fission-1 protein (Fis1), NADPH-Oxidase-2 (NOX-2), renal autophagy pathway, vascular endothelial growth factor (VEGF)/phosphatidylinositol-3-kinase (PI3K)/ protein kinase B (Akt)/endothelial nitric oxide synthase (eNOS), Nupr1/Chop/P53/PUMA/Beclin1 and Toll-like receptor (TLR)4/MyD88/TRAF6 pathways. The activation promotes pathological changes, including the disruption of the blood-brain barrier, myocardial infarction, cardiomyopathy, acute liver failure, acute kidney injury, chronic kidney disease, keratitis, retinopathy and vision loss. This review revisits the pharmacological profiles of methamphetamine and its effects on the brain, heart, liver, eyes, kidneys and endothelium. Understanding the mechanisms of methamphetamine toxicity is essential in developing treatment strategies to reverse or attenuate the progress of methamphetamine-associated organ damage.

## Introduction

The World Drug Report 2023 reported 296 million drug users in 2021, with 39.5 million categorised as a clinical drug use disorder. Amphetamines (amphetamine and methamphetamine) were the third commonly used drug, contributing to 12.2% of the overall drug users, equivalent to 36 million people. In the USA, the estimated rates of adult lifetime and past-year users of methamphetamine from 2015 to 2018 were 14.7 million and 1.6 million, respectively, with half (52.9%) of the past-year users meeting clinical use disorder criteria 1. The global geographical distribution of methamphetamine use varies, with extensive use reported in North America, Southern Africa, East and Southeast Asia, Australia and New Zealand 2.

Methamphetamine is a synthetic drug classified as an amphetamine-type stimulant together with amphetamine, methcathinone, and ecstasy-group drugs including 3,4-methylenedioxymethamphetamine (MDMA). Its clinical use is limited to relieving nasal decongestion and treating ADHD (aged ≥ 6 years) and morbid obesity to a limited extent 3, 4. The abuse potential of methamphetamine is attributable to its psychostimulant properties on the short-term general physical well-being, such as energy and sexual performance 5. The reasons for methamphetamine use in patients on opioid replacement therapy are multifactorial. One possible factor is a short-term enhancement in sexual performance to alleviate sexual dysfunction, a common adverse effect in this population 5-7.

Persistent methamphetamine use, however, leads to many deleterious effects on many organs, including the brain, heart, liver, kidneys and eyes. There is a similarity of psychopathological symptoms between certain psychiatric disorders (schizophrenia spectrum and other psychotic disorders, bipolar and other related disorders, including anxiety disorders) and some patients with methamphetamine use disorders 8-10. Moreover, methamphetamine may induce other diseases, including myocardial infarction, cardiomyopathy, acute liver failure, acute kidney injury, chronic kidney disease, hypertension, keratitis, retinopathy and vision loss. Various factors, including route of administration, dose, genetic polymorphism related to drug metabolism and polysubstance abuse, can modify the risk of methamphetamine toxicity 9. Given the significant negative impacts of methamphetamine on public health, understanding the effects of methamphetamine is crucial. This review aims to revisit the pharmacological properties of methamphetamine along with its neurotoxic, cardiovascular, hepatotoxic and ophthalmologic effects in clinical populations and preclinical models.

## Physicochemical properties

Methamphetamine exists in powder, crystal or tablet forms. Crystal meth is a physically solid, crystalline form of the drug (methamphetamine hydrochloride), appearing like shreds of glass or clear-white rocks. The crystal form can be vaporised by heating for inhalation by the users. Methamphetamine (C_10_H_15_N) and amphetamine (C_9_H_13_N) share a similar chemical structure: one aromatic ring with two carbon side chains. Both drugs contain a methyl group attached to a distal carbon side chain (alpha) of the ethylamine chain. A terminal secondary amine differentiates methamphetamine from amphetamine, which possesses a primary amine. A methyl group attached to the amine functional group compared to hydrogen in amphetamine is responsible for the enhanced lipid solubility of methamphetamine. A functional amine group in methamphetamine contributes to its base property (PKa = 9.87) 11.

**Figure 1:**
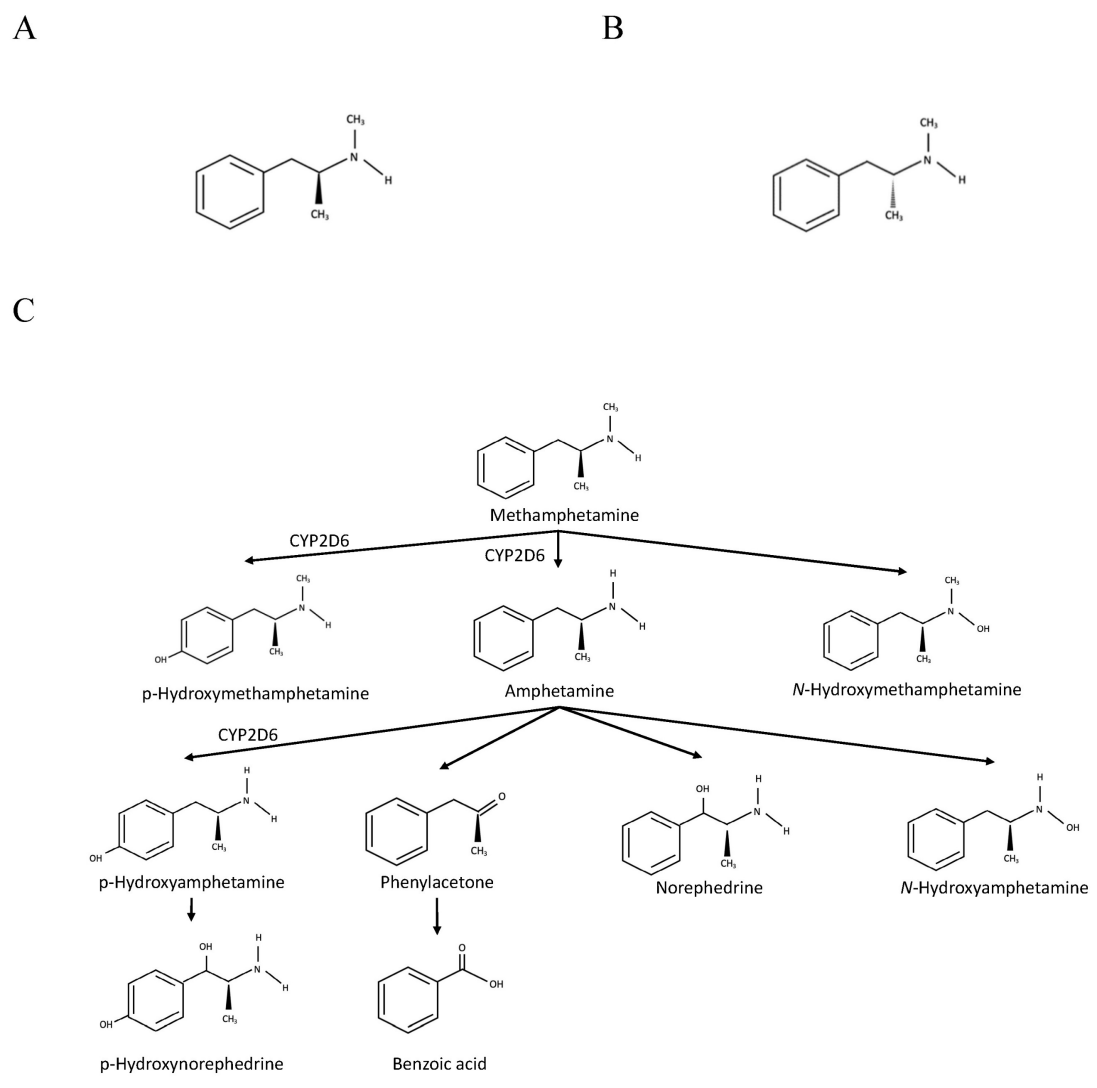
Chemical structures of methamphetamine and its products of metabolism. A. (S) (+) methamphetamine. B. ® (-) methamphetamine. C. Metabolic pathway of methamphetamine Adapted from Abbruscato and Trippier 11.

The first synthesis of methamphetamine from ephedrine took place in 1893 by Nagayoshi Nagai, followed by Akira Ogata, who first synthesised the crystallised form in 1919 4, 11. Methamphetamine has two isomeric forms: (R) (-) or l-isomer and (S) (+) or d-stereoisomer (Figure 1). The type of yield is usually determined by the methods used. Using phenyl-2-propanone (P2P) as a precursor, racemic methamphetamine can be produced by Leuckart or reductive amination methods. In the former technique, P2P reacts with formamides, producing intermediate amide, which can then react with hydrochloric acid to form racemic methamphetamine. The latter method involves an initial reaction of P2P with methylamine, aluminium and mercury chloride, forming an intermediate phenylacetone-N-methyl imine before further producing racemic methamphetamine via a reduction process. In addition, pure (S) (+) methamphetamine can be produced by reducing pseudoephedrine or ephedrine using chloride derivatives or hydroiodic acid and red phosphorus, respectively 11-13.

## Pharmacokinetics

### Route of administration and absorption

Numerous methods of methamphetamine administration have been recorded in the literature, including nasal (inhalation or insufflation), oral, intravenous, anal and vaginal routes 14. The extent of methamphetamine absorption varies, depending on the routes of administration. The earliest methamphetamine serum levels detected after oral ingestion are 1.5±0.8 hours (0.3-2.0) and 1.1±0.5 hours (0.5-2.0) for low doses (10 mg daily four times a week) and high doses (20 mg daily four times in a week), respectively 15. Meanwhile, the bioavailability for intranasal, inhalation and oral are 79%, 67%, and 67%, respectively 11, 16.

### Distribution

Methamphetamine is distributed to all body parts, including the brain, heart, lungs, liver, stomach, kidneys, eyes, hair and breastmilk 16-19. A positron emission tomography (PET) study utilising intravenous (S) (+) methamphetamine revealed that the lungs had the fastest uptake (55 seconds), one of the highest concentration peaks (22%), and the fastest clearance (7 minutes). Meanwhile, the brain had an intermediate uptake (nine minutes), one of the lowest concentration peaks (10%), and one of the slowest clearances (>75 minutes). The slow removal from the brain explains the neurotoxicity effects due to prolonged methamphetamine exposure. The heart had the fastest uptake (60 seconds), one of the lowest concentration peaks (about 3%), and intermediate clearance (16 minutes). The liver displayed the slowest uptake rate, taking 30 minutes, comparable to the stomach, and reached one of the highest concentration peaks at 23%, with clearance occurring after 75 minutes. The primary excretory organ, the kidney, required about three minutes for the uptake and had a high concentration peak based on weight (7%) and intermediate clearance (22 minutes) 19.

Following the intranasal administration of the powder type, the peak plasma concentration (t_max_) was achieved at 2.7 hours post-exposure, reaching about 113±23.1 μg/L. The smoking route demonstrated a similar t_max_ of 2.5 hours, but the peak concentration (C_max_) achieved was about half (50.9±24.7 μg/L) of that of the intranasal route 20. The distribution of methamphetamine for the oral route depends on the dose administered but typically has a lower t_max_ and C_max_ than the other two routes. For repeated low doses (10 mg daily four times a week), the drug serum levels reached a peak at 5.4±2.5 hours (2.0-8.0) with maximum concentrations of 20.2±6.4 μg/L (14.5-33.8). The high doses (20 mg daily four times a week) peaked at 7.5+3.4 hours (2.0-11.5) with highest concentrations of 32.4±7.7 μg/L (26.2-44.3). The volume of distribution after low and high doses of oral methamphetamine were 1.7±0.9 and 2.6±2.1 L/kg, respectively 15.

### Metabolism

The metabolism of methamphetamine occurs primarily in the liver. The primary cytochrome P450 (CYP) isoenzyme involved in phase I is CYP2D6, which metabolises methamphetamine via aromatic hydroxylation and N-demethylation (Figure 1). The former reaction produces p-hydroxymethamphetamine, which can be further conjugated with either glucuronide or sulphate in a phase II reaction before the urinary excretion. The N-demethylation process yields amphetamine, which can be further metabolised by CYP2D6 to form p-hydroxyamphetamine or converted into norephedrine via beta-hydroxylation 11, 21, 22.

Genetic polymorphisms of metabolising enzymes contribute to differing metabolism extent and toxicity of methamphetamine. For CYP2D6, the number and functionality of alleles determine the phenotypes for methamphetamine metabolism. Compared to CYP2D6*10, CYP2D6*1 metabolises (+) and (-) enantiomers of methamphetamine more efficiently, particularly via N-demethylation than aromatic hydroxylation 23. In a study on the Japanese population, methamphetamine abusers with intermediate metaboliser (IM-*4/*10, *5/*10, *5/*14B, *10/*10, *10/*18, *10/*36, *10/*10xn) phenotype carrying two or one reduced (*10, *14B, *10xn) and one non-functional allele (*4, *5, *14A, *18, *36) predicted lower metabolism rate than extensive metaboliser (EM) phenotype carrying one or two functional alleles (*1, *1xn, *2, *2xn) based on urine and bone marrow autopsy samples 24.

### Excretion

The main excretion route of methamphetamine is urine, eliminating approximately 37-54% of unchanged methamphetamine 18, 25. Active tubular transport plays a pivotal role in the renal excretion of methamphetamine, other than glomerular filtration clearance. The organic cation transporter (OCT)-2 and multidrug and toxin extrusion (MATE) transporters 1/2 transport methamphetamine and its metabolite amphetamine, while OCT1-3 and MATE-1 are responsible for transporting another metabolite p-hydroxymethamphetamine. The urine pH is an essential determinant for urinary excretion, with acidic pH promoting the elimination of methamphetamine and amphetamine. The reason for increased excretion is attributable to the weak base properties of methamphetamine and amphetamine, which become more ionised in the acidic environment, thus decreasing partition-mediated reabsorption. The pH-dependent MATE transporters further enhance its excretion in acidic urine pH 25.

Drug half-life and clearance are influenced by administration routes and dosage levels. Intranasal and smoking routes have the same half-life of 10.7 hours, while a 15-minute intravenous infusion of 10 mg methamphetamine shows a slightly longer half-life of 11.4 hours 20. The half-life of low oral doses (10 mg daily four times a week) is 9.3+3.7 hours (2.1-14.0), and 11.1+7.2 hours (2.2+21.2) for the high dose group (20 mg daily four times a week). The clearance of low and high doses of ingested methamphetamine are 13.2+6.5 (3.4-19.6) and 12.9+7.5 (4.5-24.7) L/h, respectively 15.

Interestingly, methamphetamine also diffuses into breast milk, which exposes danger to infants if the milk containing methamphetamine is consumed. The methamphetamine half-lives in the breast milk of two patients who smoked methamphetamine were 11.3 and 30.3 hours, which became undetectable after 100 hours of methamphetamine use. For injected methamphetamine, the predicted half-lives for two patients were 13.6 and 43.0 hours 18. Given the fact that urinary methamphetamine has an additional 30-75-hour detection window, a negative urine test for at least 24 hours can be utilised as a safety indicator for reinitiating breastfeeding 17.

Hair is another area that accumulates methamphetamine, which is eliminated from the body once the hair sheds from the scalp. The knowledge of the elimination of methamphetamine in hair is useful in detecting and monitoring drug intake in the long term. In the study by Wang and colleagues, the half-life of (S) (+) - and (R) (-) - methamphetamine ranged from 0.5-1.0 (0.6±0.1) and 0.4-0.9 (0.6±0.2) months, respectively. The former isomer predominated in hair samples, potentially due to higher content than the latter in abused methamphetamine 16. Since a fraction of methamphetamine is still detected at four months, they proposed a 6-month cut-off period to indicate abstinence from the drug after six months.

### Pharmacodynamic

One essential reason for methamphetamine abuse is the enhancement of subjective feelings of well-being, alertness and energy. Harris and colleagues 20 in their study reported increments in numerous subjective feelings, including good drug effects, drug liking, feeling high and intoxication for intranasal and smoked amphetamine with concurrent intravenous methamphetamine infusion. Notably, the intranasal route effects peaked earlier in good drug effect, feeling high and intoxication at 15 minutes. Smoked amphetamine reached the peak later at various time points: 15, 30, and 45 minutes for good drug effects, feeling high and intoxication, respectively. The earlier drug peak and earlier methamphetamine effects (good drug effects, feeling high, and intoxication) in pharmacodynamic assessment, along with higher t_max_ and C_max_ in pharmacokinetic evaluations for intranasally than smoking might partly explain the higher addictive potential of the former route 20, 26. Bad drug effects were reported in both groups, about less than 20% at peak 20. Another study discovered that acute administration of oral methamphetamine increased spatial and numeric working memory, vigilance, reaction time, and stimulant effects 27.

The enhancement of the subjective well-being of methamphetamine is attributable to its effects on monoamine neurotransmission, particularly dopamine. Other than subjective feelings, acute administration of methamphetamine produces numerous physiologic effects, such as increased blood pressure and heart rate. Harris *et al.* (2003) reported that systolic blood pressure peaked at 141 ± 13 mm Hg from 121 ± 10 mm Hg in the intranasal methamphetamine group, while the smoked methamphetamine group had a peak of 137 + 11 mm Hg with a similar baseline. For diastolic blood pressure, the pressure peaked at 86 + 7 mm Hg from 76 ± 7 mm Hg and 83 ± 8 mm Hg from 73 ± 10 mm Hg for intranasal and smoked methamphetamine groups, respectively. The heart rate was also increased in both routes of administration. The intranasal group demonstrated an increase from 73 ± 9 beats/minute to 94 ± 13 beats/minute, while the smoked amphetamine group reported an increase from 76 ± 10 beats/minute to 106 ± 19 beats/minute 20. Oral methamphetamine also has similar stimulating effects on heart rate and systolic and diastolic blood pressure 27.

## Toxicity effects

### Neurotoxicity

#### Enhancement of monoamine release into the synapse

**Figure 2:**
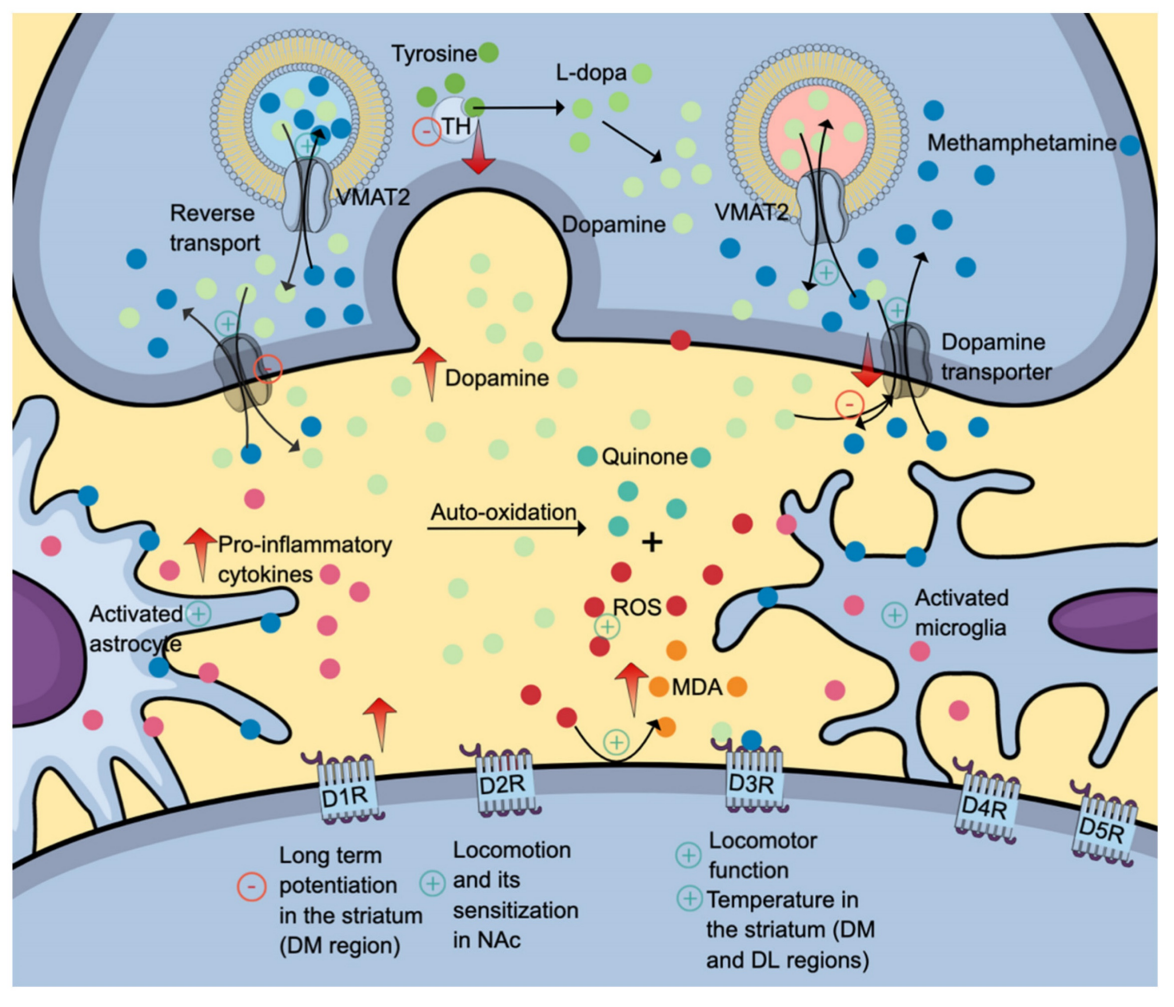
The neurotoxic effects of methamphetamine (denim blue). Methamphetamine causes a reverse transport mechanism of dopamine transporter and VMAT2, increasing the levels of dopamine (and other monoamine neurotransmitters) in the synapse. Subsequent dopamine deficit results from reduced VMAT2 and dopamine uptake into the vesicle, dopamine transporters and dopamine synthesis secondary to reduced tyrosine hydroxylase production and activity. Also, methamphetamine activates astrocytes and microglia, further contributing to neuroinflammation. Differential inhibitory and stimulatory effects of methamphetamine on various dopamine receptor subtypes might explain its effects on locomotion, learning, memory and thermoregulation. The positive symbols represent the stimulatory effects of methamphetamine, while the negative symbols represent the inhibitory effects. Abbreviations: DM: dorsomedial; DL: dorsolateral; DR: dopamine receptor; MDA: malondialdehyde (orange); ROS: reactive oxygen species (red); TH: tyrosine hydroxylase; VMAT2: vesicular monoamine transporter 2. Additional colour coding: dopamine (mint green), L-dopa (fern green), quinone (pine green), pro-inflammatory cytokines (mulberry pink), tyrosine (asparagus green).

The high lipophilic property of methamphetamine allows a simple diffusion of drug across the blood-brain barrier. In the brain, methamphetamine can diffuse into the nerve terminals via monoaminergic transporters owing to its shared chemical structure with monoamines (dopamine, serotonin, and norepinephrine). Given its weak base characteristics, methamphetamine accumulates and alkalinises intracellular environment (Figure 2). The intracellular alkalinisation affects the uptake and accumulation of neurotransmitters in storage vesicles, which depends on the pH gradient between the internal and external sides of the vesicles. Methamphetamine can also accumulate in the vesicles. These intracellular effects prevent the uptake of monoamines into the vesicles and cause the release of monoamines into the cytosol (via vesicular monoamine transporter 2 (VMAT2) and synapse (via monoamine transporters) by a reverse transport mechanism. Interestingly, its effect on VMAT2 is not attributable to the inhibition of ATPase but rather the reversal of the transporter. Excessive accumulation of neurotransmitters in the synapse is also attributable to methamphetamine effects on neurotransmitter uptake and metabolism 4, 28, 29. The presence of high levels of neurotransmitters stimulates various monoamine receptors located in the postsynaptic membrane. Increased dopaminergic neurotransmission is the mechanism for reward, learning and memory. Serotonin regulates impulsivity, stress, mood, learning and memory, whereas norepinephrine promotes arousal and attention, as well as regulates stress, mood, learning, and memory 4.

#### Effects on dopaminergic neurotransmission in numerous brain regions

Dose and duration are two determining factors contributing to methamphetamine effects on dopaminergic neurotransmission. At a low concentration, acute administration of methamphetamine induces dopaminergic neuronal firing via dopamine transporter-mediated excitation and synaptic transmission. In contrast, methamphetamine reduces cell firing at high concentrations via dopamine D2 auto-receptor activation, evidenced by the reduction of dopamine inhibitory postsynaptic current amplitudes 30. Regarding the duration, acute binge-like dosing produces an increase in dopamine levels, while prolonged administration produces the opposite effects. A study utilising a rodent model reported a significant elevation in dopamine levels within the frontal cortex (at 2- and 24-hours post-treatment) and amygdala (at 24 hours post-treatment) following one-day binge-like dosing of methamphetamine. Interestingly, a decrease in 3,4-dihydroxyphenylacetic acid (DOPAC) levels at 24- and 48-hour post-treatment was observed in the striatum, indicating a reduction in dopamine turnover 31.

The dopaminergic deficit in the striatum is attributable to reduced VMAT2 and dopamine uptake into the vesicle, dopamine transporters and dopamine synthesis secondary to reduced tyrosine hydroxylase production and activity (Figure 2) 32. Methamphetamine enhances dopamine release when administered acutely. Upon secretion, dopamine binds to its receptors, initiating a cascade of diverse cellular, molecular and behavioural changes. All dopamine receptors (D1R-D5R) play various roles in drug addiction and relapse, including methamphetamine-induced behavioural sensitisation. Interestingly, the dopamine D3 receptor (D3R) exerts a positive regulatory effect on methamphetamine-induced locomotor function but not the basal spontaneous motor activity which is regulated by other dopamine receptors 33.

Further, binge methamphetamine use decreases the striatal dopamine transporter (DAT) (Figure 2) uptake in dorsolateral and dorsomedial areas, which coincides with the increase in body temperature 34, 35. Repeated injections of a specific dopamine D3R antagonist, PG01037, mitigate the effects of methamphetamine on DAT and body temperature. These findings proposed the role of D3R in methamphetamine-induced hyperthermia, a well-documented phenomenon contributing to neuronal damage 34, 35. Interestingly, similar effects of PG01037 on the striatal DAT uptake are not replicable in warmer and more ambient conditions, suggesting a complex interaction between intrinsic and environmental factors 34. Another methamphetamine-induced neurotoxic effect in the striatum is impaired long-term potentiation in the dorsomedial region mediated by D1R. The attenuation of long-term potentiation might explain the neurotoxic effects on memory and learning 34.

Nucleus accumbens (NAc) is another area affected by methamphetamine use, evidenced by reduced expressions of D1R and D2R 33. D1R- and D2R-medium spiny neurons (MSNs) are the primary neurons found in the NAc. The activation of the former neurons facilitates drug-seeking behaviour, while the latter causes the opposite effects. D2R-MSNs project to the ventral pallidum, which is connected to the ventral mesencephalon. The activation of the D2R-MSNs-ventral pallidum-ventral mesencephalon pathway inhibits the thalamus and, thus, drug-seeking behaviour (indirect pathway). The updated concept informed that D2R-MSNs can also directly cause inhibition through the D2R-MSNs-ventral pallidum-thalamus pathway 36. The NAc-specific D2R-knockdown mice have been reported to exhibit reduced acute methamphetamine-mediated hyperlocomotion and attenuated the extent of locomotor sensitisation upon repeated methamphetamine dose. These findings further support the role of D2R in regulating motivated and addictive behaviours 37.

#### Oxidative stress and inflammatory response

Methamphetamine induces neurotoxicity in various brain areas attributable to impaired dopaminergic and serotonergic circuits, neuronal apoptosis and neuroinflammation induced by astrocytes and microglia activations (Figure 2). Several studies reported that activation of neuronal nitric oxide synthase (NOS) promoted overproduction of nitric oxide radicals (such as peroxynitrite), contributing to oxidative stress 38. Other than dopamine, methamphetamine also induces glutamate release. The binding of glutamate to NMDA receptors causes calcium influx and activation of neuronal NOS. Activated neuronal NOS catalyses the generation of nitric oxide which can react with superoxide, forming peroxynitrite 38. Several inhibitors of nitric oxide synthase, such as 7-nitroindazole (7-NI), *N*^G^-nitro-L-arginine and *N*^G^-nitro-L-arginine methyl ester have been reported to attenuate methamphetamine-induced dopaminergic neurotoxicity, suggesting the essential role of nitric oxide in the pathophysiology of methamphetamine neurotoxicity 38, 39. Interestingly, the results from clinical studies on the central effects of methamphetamine on glutathione, a primary antioxidant, are equivocal. One study reported that methamphetamine use disorder patients had a significantly higher level of glutathione (GSH) in the dorsolateral prefrontal cortex compared to healthy controls 40. In contrast, another study found negligible differences between the two groups despite higher peripheral immunoinflammatory markers in patients than in healthy controls 41.

### Cardiotoxicity

Many studies have reported the cardiotoxicity effects of methamphetamine, leading to numerous diseases, such as coronary heart disease, arrhythmias, myocardial infarction, cardiomyopathy and heart failure 10. Further, methamphetamine use is not only associated with various heart-related illnesses but also accelerates cardiovascular diseases (CVD) a few years earlier than the typical CVD onset 42-44. Compared to CVD patients without a history of methamphetamine use, those using methamphetamine had worse dysfunction in various parameters, including left ventricular (LV) ejection fraction (EF), LV mass index, end-diastolic volume (EDV) index and myocardial perfusion 43-45. The risk factors for methamphetamine-associated CVD development include hypertension, chronic kidney disease, diabetes and smoking 10. Mechanisms of methamphetamine-induced cardiotoxicity are described in detail below.

#### Oxidative stress

**Figure 3:**
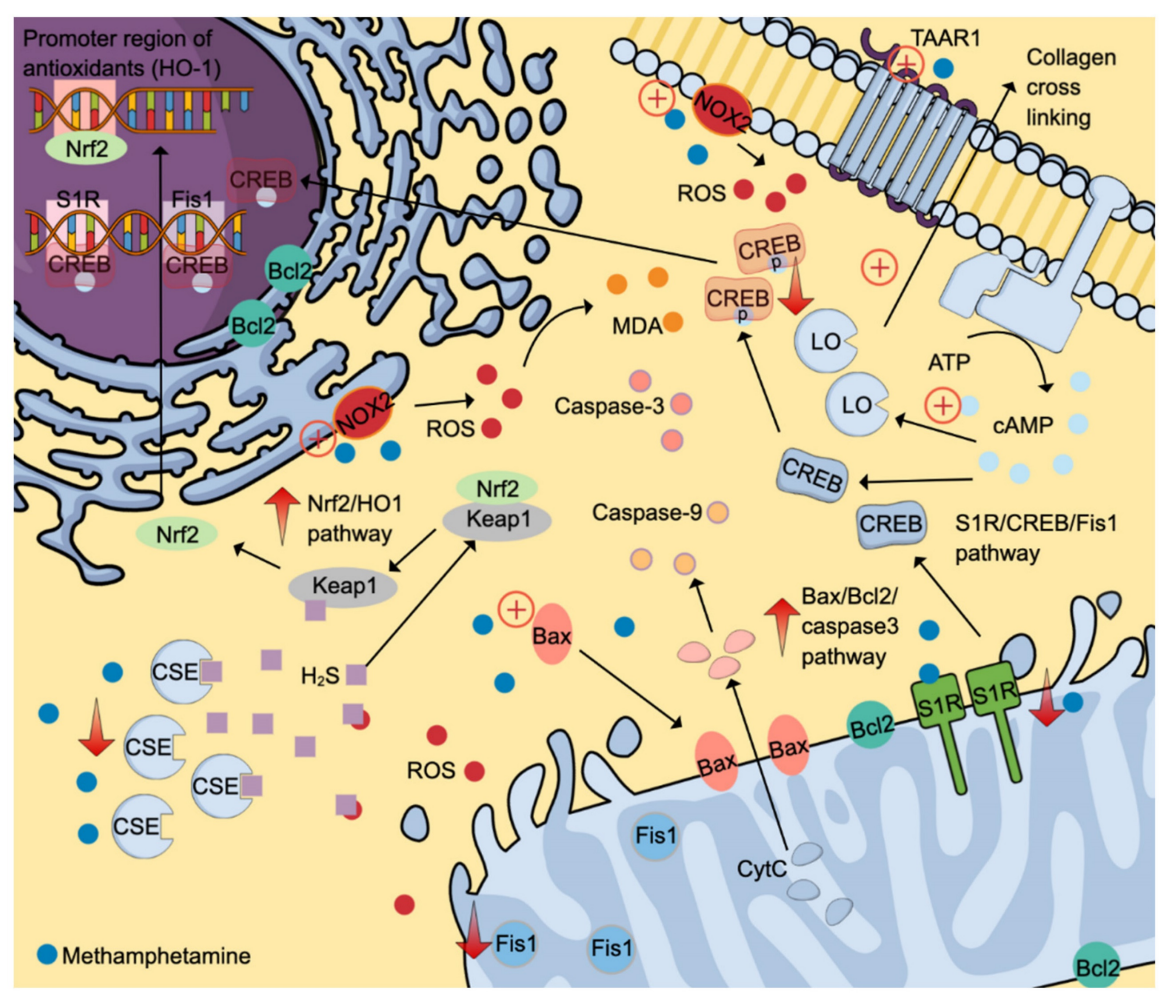
Molecular-signalling pathways activated by methamphetamine contribute to cardiotoxicity. Methamphetamine induces ROS formation by stimulating NADPH oxidases 2 to generate ROS. ROS then causes lipid peroxidation and activates S1R/CREB/Fis1 and Nrf2/HO1 pathways. Also, methamphetamine promotes cell apoptosis by stimulating the Bax/Bcl2/caspase-3 pathways. Fibrotic-inducing properties of methamphetamine are mediated through the TAAR1/cAMP/LO pathway. The positive symbols represent the stimulatory effects of methamphetamine (denim blue), whereas the negative symbols represent the inhibitory effects. Abbreviations and colour coding: cAMP: cyclic adenosine 3,5-monophosphate (Uranian blue); CSE: cystathionine gamma-lyase; CREB: cAMP response element-binding protein; CytC: cytochrome C (baby blue); Fis1: mitochondrial fission-1 protein; HO-1: heme-oxygenase-1; H_2_S: hydrogen sulfide (pastel purple); keap1: Kelch-like ECH-associating protein 1; LO: lysyl oxidase; MDA: malondialdehyde (orange); NOX: NADPH oxidases; Nrf2: nuclear factor-erythroid factor 2-related factor 2; ROS: reactive oxygen species (red); S1R: Sigmar 1, TAAR1: trace amine-associated receptor 1. Additional colour coding: Caspase-9 (light orange), caspase-3 (salmon pink).

Evidence from animal studies found that methamphetamine can induce oxidative stress in the cardiovascular system as evidenced by the elevation of a reactive oxygen species (ROS) superoxide and a lipid peroxidation product, malondialdehyde (MDA) 46, 47. NADPH oxidases (NOX) are primary ROS generators, and methamphetamine has been shown to stimulate catalytic subunits (p47^phox^ and gp91^phox^) of NOX2 (Figure 3) 47. An inhibitory effect of methamphetamine on cardiomyocyte mitochondrial electron transport chain via suppression of the Sigmar1/cyclic adenosine 3,5-monophosphate (cAMP) response element-binding protein (CREB)/mitochondrial fission-1 protein (Fis1) pathway is also partly attributable to oxidative stress (Figure 3) 48.

Oxidative stress leads to the depletion of various endogenous antioxidants, including superoxide dismutase and hydrogen sulphide (H_2_S). The reduction of H_2_S is partly attributable to reduced production and excessive use to fight against oxidative stress. The former cause is potentially due to reduced cystathionine gamma-lyase (CSE) expression, one of the primary H_2_S-producing enzymes 47. H_2_S can neutralise various ROS, including hydrogen peroxide, superoxide, and peroxynitrite. Further, H_2_S upregulates nuclear factor-erythroid factor 2-related factor 2 (Nrf2)-signalling pathway by interacting with the Kelch-like ECH-associating protein 1 (Keap-1), which is attached to Nrf2 in the cytoplasm. The release of Nrf2 allows its translocation into the nucleus to activate various promoter regions responsible for antioxidant system activation (Figure 3) 49.

Interestingly, methamphetamine exposure promotes the expression of Nrf2, which in turn induces the heme-oxygenase-1 (HO-1) promoter region. The activation of the Nrf2/HO-1 pathway exerts cytoprotective effects, attenuating oxidative stress by promoting antioxidant effects 46. Besides oxidative stress, methamphetamine also promotes apoptosis via the Bax/Bcl2/caspase-3 pathway (Figure 3) 46.

#### Inflammatory response

Regarding inflammation, cardiac transcriptional analysis in the animal models reported upregulations of various inflammation-related genes, including Bpifa/Plunc, RegIIIg and Scgb3a2 50. Together, oxidative stress, inflammation and apoptosis effects of methamphetamine lead to structural and functional abnormalities.

#### Fibrotic, vascular, structural and functional effects

One of the most common structural changes induced by long-term methamphetamine administration is cardiac fibrosis, which is observed in various animal models (zebrafish and mice) and humans 46-48, 51, 52. Fibrotic histopathological changes observed in preclinical and clinical samples include excessive collagen deposition and elevated fibrotic markers of periostin and smooth muscle actin 48, 50. Methamphetamine-induced fibrotic properties are partly attributable to its ability to stimulate G-protein-coupled receptor (GPCR) trace amine-associated receptor 1 (TAAR1), which utilises a cAMP second messenger system in cardiomyocytes. Elevated cAMP levels then promote lysyl oxidase (LO) production, an essential enzyme that forms crosslinking between collagens in the extracellular matrix (Figure 3) 51. Other histopathological changes include nucleolysis, nuclear fission and hyperfused and extended mitochondrial networks 46, 48. Clinical findings reported in the literature include intracardiac thrombus and elevated LV mass 52. Histopathological findings of autopsy cases of methamphetamine revealed various cardiac pathologies, such as myocardial fibre hypertrophy, myocardial infarction, interstitial and perivascular fibrosis, atherosclerosis (mild to severe), congestion and focal degeneration/necrosis 53.

Molecular and structural changes observed are parallel to elevated biochemical markers of cardiac injury, including creatine kinase MB (CKMB), lactate dehydrogenase (LDH) and cardiac troponin I (cTnI) 46. These pathological changes can cause disturbances in cardiac function. Cardiac dysfunction associated with methamphetamine use varies in pre-clinical models, ranging from reduced LVEF, reduced fractional shortening (FS) and increased LV internal diameter (LVID) systole and diastole 47. In clinical studies, similar cardiac dysfunctions were reported, including elevated end-systolic/diastolic volumes, reductions in ventricular EF and reduced coronary microcirculation 43, 45, 52.

#### Sex-specific effects

Methamphetamine causes sensitisation effects as early as nine days in a rodent model characterised by elevated heart rate and EF but reduced stroke volume 54. Although methamphetamine-cardiotoxicity effects are sex-dependent, various pre-clinical models reported inconsistent results. Marcinko *et al.* 50 reported that only male mice developed dilated cardiomyopathy at five months of methamphetamine exposure, characterised by reduced EF and FS but elevated LVID at systole and diastole. Interestingly, the mortality rate for male mice was more than 50% compared to none in female mice.

Contradictory, a 10-day methamphetamine exposure caused prominent changes in circadian clock gene expression in only female rats, upregulating *Per2* and* Per3* while downregulating *BMal1*, *Npas2 and Clock* genes. Circadian clock genes play a pivotal role in the diurnal regulation of blood pressure, heart rate, and heart metabolism. Altered expression of these genes can increase the risk of cardiovascular disease. Interestingly, one-month abstinence from methamphetamine reversed the altered circadian clock genes 55. Another study utilising the same treatment protocol in addition to an ischemic-reperfusion period also displayed a higher susceptibility of female rats to methamphetamine toxicity effects. More extensive post-ischemic infarct size and contractility dysfunction at systolic and diastolic were reported in only female rats after a 10-day treatment as well as a 10-day-and-one-month abstinence 56. Different treatment protocols in dosing (constant vs increasing), duration (ten days vs five months), frequency (daily vs week and administration route (subcutaneous vs intraperitoneal) might partly explain the differing outcomes between studies.

#### Electrophysiological effects

A study using a zebrafish model exposed to methamphetamine demonstrated unexpected outcomes in electrophysiological aspects 51. Heart rate was reported to reduce 51 in contrast to other preclinical and clinical findings 45, 54, 57, 58. The possible explanation is that elevated blood pressure caused by methamphetamine stimulates a baroreceptor reflex, leading to reduced heart rate. An increase in heart rate variation (HRV) during the first week of methamphetamine exposure might also be attributed to a similar mechanism. Interestingly, Zhang and colleagues 51 found a reduction in the HRV of zebrafish exposed to methamphetamine at week two, possibly due to persistent inflammation and cardiac injury. Another interesting finding is a reduced QTc interval, a contradictory phenomenon observed in clinical cases. The possible reason for this condition is attributable to early changes caused by methamphetamine before overt cardiomyopathy is established 51.

#### Cardiovascular changes following abstinence

Regarding cardiovascular effects following abstinence, a preclinical model of rhesus macaques on long-term methamphetamine (3.6-8.6 years) provided some insight into the dynamic changes of the heart. In the study, researchers found tolerance effects of methamphetamine on diastolic pressure (day 1) and systolic pressure (day 1 and week 1) post-abstinence and sensitisation effects on diastolic pressure (week 12) and heart rate (week 26). Other functional effects included reductions in EF and cardiac output (CO). Interestingly, the abnormal physiological measures returned to control values in one year, indicating the reversibility of the methamphetamine-induced cardiac effects 58. In a clinical population, a complete cessation of methamphetamine improved EF and heart failure-related hospital admission in heart failure patients with reduced EF 44.

#### Effects of prenatal exposure

Another essential area is the prenatal exposure effect of methamphetamine on the heart in adults. In the pre-clinical model, methamphetamine exposure during the first half, second half, or whole pregnancy period increased the infarct size following an episode of ischemic-reperfusion injury in adult female rats. No significant differences were reported between treatment exposures, indicating that full abstinence is required to avoid the cardiac sensitisation effect of methamphetamine. In contrast, the authors reported no significant effects of prenatal methamphetamine exposure on functional parameters, including heart rate, developed pressure, +dP/dT and -dP/dT 57.

Other effects of prenatal methamphetamine exposure in an adult animal model are the long-term epigenetic changes. Females are more susceptible to cardiac epigenetic changes. Dague and colleagues 59 in their study reported that female adult rats exposed to methamphetamine exposure during a prenatal period exhibited two times higher non-similar gene expressions. Two primary changes were observed in dimethylarginine dimethylaminohydrolase-2 (DDAH2) and 3-hydroxybutyrate dehydrogenase 1 (BDH1). Interestingly, the protein expression of BDH1 was also reduced in adult rats of both sexes. BDH1 is particularly useful as an energy production using ketones in pathological conditions, including cardiac hypertrophy and heart failure. Reduced expression is associated with cardiac dysfunctions 59. In contrast, although reduced gene expression of DDAH2 was observed in both sexes, only female rats reported a decrease in protein expression, suggesting a possible compensatory mechanism in male rats. DDAH2 degrades asymmetric dimethylarginine which is a nitric oxide synthase inhibitor. The effect of methamphetamine on DDAH2 expression can potentially cause asymmetric dimethylarginine accumulation and vascular dysfunction 59.

### Hepatotoxicity

The liver is among the most susceptible organs to methamphetamine toxicity. The extent of methamphetamine-induced hepatotoxicity ranges from mild liver injury to severe fulminant hepatic impairment. Pathological changes of hepatic complications include fibrosis, necrosis, ballooning degeneration in centrilobular zones, hepatomegaly and hepatitis. Further, methamphetamine's effect on the liver is associated with other health issues, such as cognitive deficits and psychiatric symptoms 60.

The liver hosts several transaminases essential for synthesising and degrading amino acids and converting energy-storing compounds. The levels of transaminases are usually low but increase in liver injury, secondary to increased cell membrane permeability, allowing intracellular enzymes to escape into the blood. Numerous preclinical studies reported that methamphetamine exposure led to increased blood levels of alanine transaminase (ALT), aspartate transaminase (AST), alkaline phosphatase (ALP) and ammonia compared to the control group 61-66. Also, patients with methamphetamine use disorder had significantly higher ALT and AST levels compared to healthy controls 67. The biochemical changes indicative of liver injury are parallel to histopathological findings observed in methamphetamine users (Eskandari *et al.* 2014; Wang *et al.* 2017). These changes include nuclear abnormalities (shrinkage, karyopyknosis and nuclear migration), cytoplasm damage (loosening, vacuolar degeneration, microvesicular lipid and increased glycogen), cellular changes (hydropic change and hypertrophy), mitochondrial aggregation, and inflammatory cell infiltration in the portal and lobular areas 61-64, 66, 68, 69. In contrast, Azizi *et al.* (2023) 70 reported vascular degeneration and congestion not only in the exposed group but also in the control group. The inconsistent findings might be attributable to different study designs, with various rodent types and dose and duration of methamphetamine exposure.

The precise mechanisms underlying methamphetamine-induced liver damage remain unclear. Potential factors contributing to methamphetamine toxicity are hyperthermia, disruption of the CYP1A2 metabolic pathway, oxidation of biogenic amines, hyperammonaemia, mitochondrial impairment, apoptosis, inhibition of cell division, elevated neurotransmitter efflux and the impairment of bile acid homeostasis by gut microbes 64, 66, 71, 72. The following subsections further discuss on the mechanism of methamphetamine-induced hepatotoxicity.

#### Oxidative stress

ROS formation induced by methamphetamine elicits pathological downstream events, such as lipid peroxidation and antioxidant depletion (glutathione peroxidase (GPx), superoxide dismutase (SOD)), promoting oxidative stress 63.

#### Inflammatory response

Oxidative stress activates a toll-like receptor (TLR)4/MyD88/TRAF6 pathway, promoting inflammation 61, 64, 68. TLRs play a key role in regulating innate and adaptive immunity, pathogen detection and inflammatory signalling (Lai *et al.*, 2015; Xie *et al.*, 2018). Activated adaptor proteins MyD88 and TRAF6 by TLR4 trigger downstream inflammation (Xie *et al.*, 2018), promoting the expression of various inflammatory markers, including TNF-α, IL-6, IL-1β, and IL-18 61, 64, 66, 68. Methamphetamine-induced cell cycle arrest and apoptosis in hepatocytes may result from the disruption of essential cellular bioprocesses 65.

In a clinical population, a study examining the association of methamphetamine use with liver pathology in autopsy samples of 527 (413 cases and 114 controls) reported similar occurrences of fatty liver and cirrhosis in both groups. Hepatitis and infiltration of inflammatory cells (lymphocytes and plasma cells) in the portal triads (areas of the liver that contain bile ducts, veins and arteries) were significantly higher in cases but no significant differences were observed between methamphetamine and other intravenous drug users 73. The exact reason for negligible differences between groups is unknown, but potentially due to other factors such as shared pathological changes in intravenous drug users regardless of the type of drugs and inclusion criteria for the control group (drug-free (based on the post-mortem toxicological exam) trauma victim).

#### Vascular effects

A case report from another autopsy finding of a case of a 35-year-old man due to methamphetamine overdose revealed multi-organ ischemic insults, secondary to methamphetamine-induced vasoconstriction in the liver, heart (left ventricle) and brain (cerebellum). Histopathological examination revealed necrotic hamartoma in the liver and pancreas and extensive fibrosis and thrombi in the liver. Also, fat necrosis with infiltration of acute inflammatory cells, oedema and fibrinous exudate were observed in the pancreas 74.

#### Bile acid profile derangements

The effect of methamphetamine on the liver can disrupt bile acid production, leading to decreased bile acid production and deficiency in secondary bile acids. Ma and colleagues 67 conducted a study to investigate the association of various parameters concerning lipid profile, bile acids and psychiatric psychopathology in methamphetamine use disorder patients during the withdrawal period. The effects of methamphetamine were the most prominent at a 3-month withdrawal point when patients showed the greatest derangement in transaminases (AST and ALT), lipid parameter triglyceride and psychiatric symptomatology of depression and anxiety. Interestingly, the bile acid profiles, including total, primary (CA and CDCA) and secondary bile acids (HCA and UDCA), were significantly lower in patients than in HC. The findings suggest the role of bile acids as potential mediators of liver injury and psychiatric comorbidities in methamphetamine withdrawal, which could be linked to disease progression, potentially through the crosstalk between the liver and brain 67. The association between liver injury and mental health problems or cognitive impairment has long been established, particularly in patients with non-alcoholic fatty liver disease (NAFLD). NAFLD patients possess a higher risk of cognitive impairments (memory, visual attention and coding ability) and depression 75, 76.

### Ocular toxicity

A range of ophthalmologic complications in methamphetamine use disorder patients has been reported in the literature. The methamphetamine ophthalmologic-related sequela includes keratitis, endophthalmitis, vision loss, retinopathy, intra-retinal haemorrhages, retinal vasculitis, amaurosis fugax, optic neuropathy and acute closed-angle glaucoma 77-81.

#### Local or direct effects

**Figure 4:**
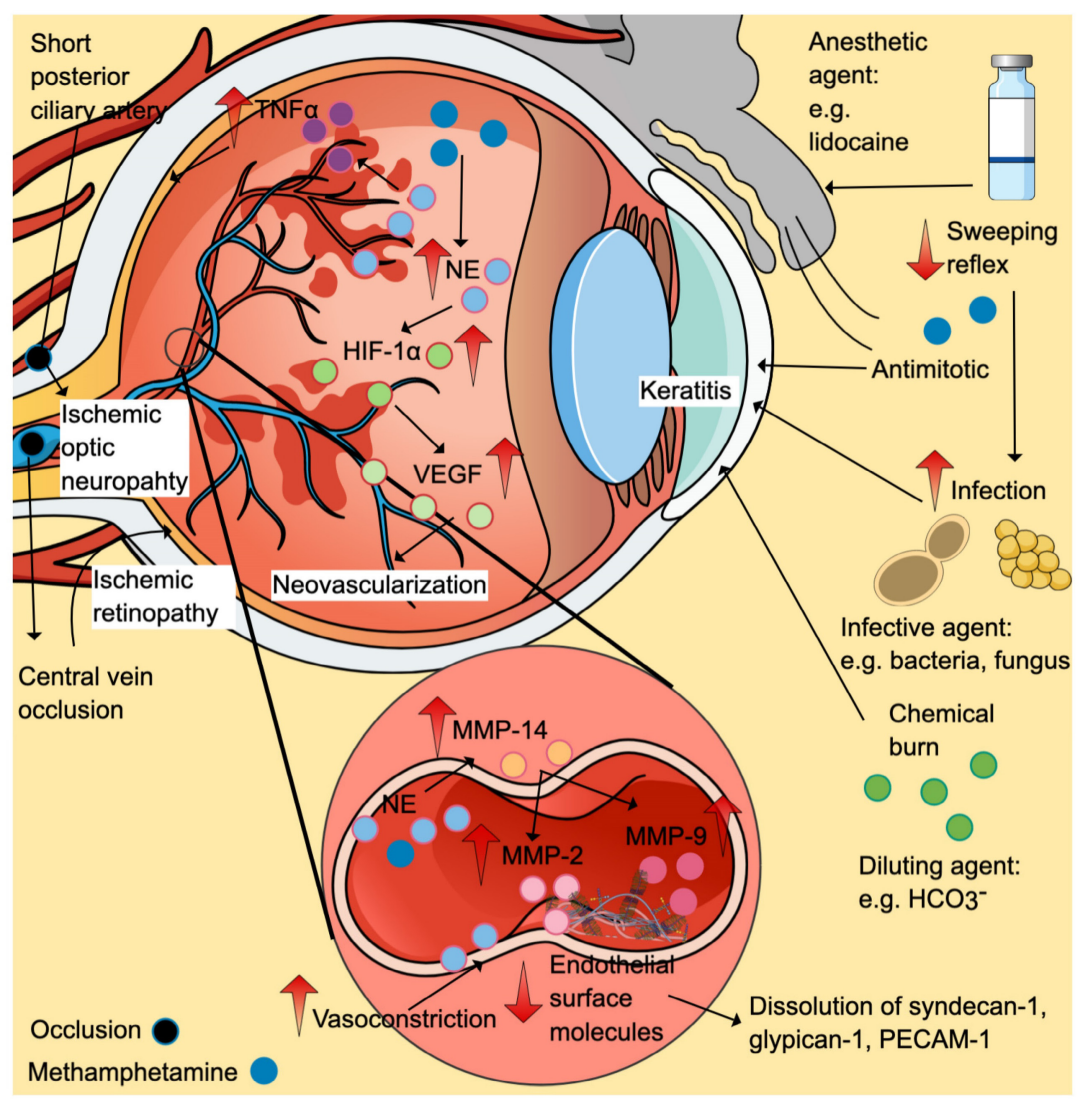
Ophthalmologic effects of methamphetamine. Methamphetamine can cause direct effects (chemical burn), reduced sweeping reflex (may increase the risk of infection), oxidative stress, inflammation (↑ TNF-α), vasoconstriction (may increase the risk of retinopathy), neovascularisation (secondary to ischemic-induced increased in HIF-1α/ VEGF), and dysregulation of extracellular matrix proteins (through MMPs). The positive symbols (and colour) represent the stimulatory effects of methamphetamine (denim blue), while the negative symbols represent the inhibitory effects. Abbreviations: HIF-1α: hypoxia-inducible factor-α (fern green); MMP: matrix metalloproteinases; NE: norepinephrine (baby blue); PECAM-1: platelet endothelial cell adhesion molecule-1; TNF-α: tumor necrosis factor-α (purple); VEGF: vascular endothelial growth factor (mint green). Additional colour coding: asparagus green (diluting agent), lace pink (MMP-2), mulberry pink (MMP-9), and orange (MMP-14).

Keratitis is the commonest case reported, partly attributable to chemical irritation, vasoconstriction (secondary to catecholamine release), decreased blink reflex, scratching and self-epilation exacerbations, increased infection susceptibility and elevated pain threshold. Direct contact with methamphetamine vapour can irritate the eyes (Figure 4). Given the close location between the eyes and nasopharynx, inhaled methamphetamine can also cause local toxic effects. Accidental introduction of methamphetamine and its diluting agents when the contaminated hand touch the eyes is another possible cause. Diluting agents like bicarbonate can cause chemical burns 82.

#### Suppression of weeping and blinking reflexes

Besides, anaesthetic agents in diluting solution (including lidocaine) can suppress weeping and blinking reflexes, reducing corneal protective ability while increasing infection risks. Infective agents such as gram-positive (Staphylococcus aureus, Streptococcus spp. and Propionibacterium acnes) and negative (Pseudomonas and Capnocytophaga) bacteria and fungi (Candida albicans) have been isolated from the sensitivity tests of keratitis cases 77, 83, 84. Although infections are commonly found, keratitis with no evidence of infection has also been previously reported 82. The agent can also disturb the re-epithelialisation via its anti-mitotic effects and disruption in cellular respiration 83, 85.

#### Vascular effects

Vascular effects via sympathomimetic effects of methamphetamine can cause vasospasm and vasoconstriction, leading to ischemic retinopathy and ischemic optic neuropathy. Increased plasma levels of norepinephrine, a potent vasoconstrictor, have been reported in methamphetamine animal models (Figure 4) 86. In the eyes, the short posterior ciliary arteries are the primary blood supply to the optic nerve head (optic disk). The ischemic episodes can cause ischemic optic neuropathy 79. Ischemic retinopathy cases have been reported in methamphetamine users involving occlusions of the central retinal vein and bilateral branch retinal arteries 87, 88. Ischemic insults favour the formation of hypoxia-inducible factor 1α (HIF-1α), which in turn upregulates the expression of vascular endothelial growth factor (VEGF). Retinal neovascularisation is the consequence of HIF-1α-induced VEGF elevation, as seen in clinical cases and preclinical studies 87, 89.

#### Extracellular matrix protein dysregulation

Apart from the vasoconstrictive effects of norepinephrine, its excessive levels increase metalloproteinase (MMP)-14, which promotes the activation of MMP-2 and MMP-9, dissolving numerous endothelial surface molecules such as syndecan-1, glypican-1 and platelet endothelial cell adhesion molecule-1 (PECAM-1) (Figure 4) 86.

#### Oxidative stress and inflammatory response

Excessive norepinephrine promotes a pro-inflammatory state, evidenced by the increase in tumour necrosis factor-alpha (TNF-α) and oxidative stress markers in the retina and blood 90, 91. The TNF-α cell death-signalling pathway and oxidative stress might partly explain retinal neurodegeneration as shown by thinning retinal nerve fibre layer thickness (RFNL) and a reduction in Bruch's membrane opening minimum rim width reported in clinical and animal studies 86, 90, 92. Other pathologies include marked activation of astrocytes and microglia secondary to methamphetamine exposure 93.

#### Talc deposition

Talc deposition in the macula is another form of retinopathy known as crystalline retinopathy. Kumar, Kaiser 94 reported a case of crystalline retinopathy in a patient who had been snorting methamphetamine for years. Although talc deposition is more common in patients who administer crushed oral drug suspension intravenously, nose or lung are possible routes of delivering talc to retinal circulation 94.

#### Neural effects

Another pathological change induced by methamphetamine is the abnormality in the sensory receiving area and visual pathway, which might cause a delay in signal delivery due to altered processing in the primary visual area 78.

### Renal toxicity

Methamphetamine can cause acute kidney injury (AKI) and chronic renal disease (CKD) 95-98. Acute manifestations of methamphetamine-induced AKI may include acute tubular necrosis (the most prevalent), tubulointerstitial nephritis and thrombotic microangiopathy 96, 99-102. Hyperthermia, rhabdomyolysis, tubular obstruction, vasoconstriction and hypovolemia are possible causes of AKI secondary to methamphetamine intoxication 101.

#### Thermoregulatory effects

The effect of methamphetamine on serotonin and dopamine levels can lead to hyperpyrexia, one of the contributing factors of rhabdomyolysis 34, 35, 100. As previously stated, D3R stimulation by methamphetamine causes hyperthermia 34, 35. Hyperthermia is one of the common signs reported in rhabdomyolysis cases, particularly in severe methamphetamine intoxication 96, 102. Further, elevation of 70 kDa heat shock protein, a protective molecule against heat stress, in myoglobin-positive kidneys of methamphetamine users may support the role of this protein in the pathophysiology of hyperthermia and AKI secondary to methamphetamine intake 102.

#### Rhabdomyolysis

Rhabdomyolysis is a common manifestation in acute methamphetamine intoxication, accompanied by excessive release of creatine kinase from damaged skeletal muscle. A meta-analysis study involving acute methamphetamine intoxication cases reported that the weighted mean values of five studies were seven times higher than the normal upper limits of creatine kinase 103. The creatine kinase levels with a cut-off point of 10,000 IU/l in acute poisoning cases may be a useful predictive tool for AKI (84% sensitivity, 69% specificity) 104.

Other than creatine kinase, myoglobin released from injured skeletal muscle is another essential biomarker. Rhabdomyolysis induces the release of myoglobin into the systemic circulation, which is subsequently deposited in the kidney 105. The iron component (heme protein) of myoglobin can undergo redox cycling between ferric and ferryl forms, initiating lipid peroxidation reactions. Potent renal vasoconstrictors, F_2_-isoprostanes, are one of the lipid peroxidation products responsible for renal vasoconstriction 106. Furthermore, F_2_-isoprostanes can stimulate endothelin-1 production (another potent vasoconstrictor) 107. In the kidney, endothelin-1 is produced primarily by endothelial and tubular cells. Overactivity of the endothelin-1 type A over type B receptors may be responsible for developing renal disease 108. Interestingly, urinary alkalinisation reduces urinary F2-isoprostane levels and improves renal function, potentially owing to a decreased ferryl myoglobin reactivity 106.

#### Oxidative stress

Myoglobin also increases other oxidative stress markers, including 8-hydroxy-2' -deoxyguanosine (an oxidative DNA damage marker), 4-hydroxy-2 -nonenal (a product of lipid peroxidation) and superoxide dismutase (a free radical scavenger) in a clinical population 102. In a preclinical study, Zhang *et al.* reported increased oxidative stress in animals exposed to methamphetamine, evidenced by increased GSH levels and GPx activities, besides reduced levels of MDA and protein carbonyls and activities of catalase 98, 109. Interestingly, pre-treatment with pharmacological agents with antioxidant properties, such as caffeic acid and *N*-acetylcysteine amide, confer protection against methamphetamine-induced oxidative stress 98, 109, 110. However, the exact causes (either direct methamphetamine toxicity or other factors, such as myoglobin) cannot be concluded, as the study did not assess the presence of myoglobin casts in the kidney 109, 110. Two potential pathways activated by methamphetamine use are the Nrf2/HO-1 pathway, which enhances antioxidant effects, and the renal autophagy pathway, which stabilises cellular homeostasis by degrading damaged macromolecules 111.

Oxidative stress and inflammation caused by methamphetamine can induce various pathological changes. Histopathological changes of renal biopsy in methamphetamine intoxication secondary to rhabdomyolysis (confirmed/suspected) include myoglobin-positive granular casts in the distal tubules, detached renal epithelium in the lumen and interstitial oedema of the renal medulla in clinical populations 96, 100, 102. Interestingly, myoglobin casts are not always detected, with the detection rate ranging from 19-66% in the most prevalent renal toxicity findings (ATN), suggesting that other pathophysiologies might be essential for AKI among methamphetamine abusers 99, 102.

#### Tubular obstruction

Intratubular myoglobin in the kidney can react with Tamm-Horsfall protein in acidic urine, forming intratubular casts. These casts may block the renal tubular system, contributing to AKI 70, 112. Hypovolemia, acidosis and ischemia are other contributing factors that can worsen AKI 112.

#### Volume depletion

Isoardi and colleagues 101 in their prospective observational study reported 90% presentations of AKI in the Emergency Department among methamphetamine intoxication patients with abnormal creatinine levels. The incidence of rhabdomyolysis was almost half (44%) among 50 presentations at the Emergency Department 101. Most AKI presentations were mild in severity and successfully attenuated by crystalloid therapy. The possible reasons for volume depletion-induced AKI in this situation are attributable to concurrent psychomotor agitation and reduced fluid intake 101.

#### Repeated renal insults

Persistent renal insults secondary to chronic methamphetamine abuse and other associated factors (including malignant hypertension and uncontrolled diabetes) can increase the risk of CKD. Chronic renal pathologies associated with methamphetamine consumption include tubulointerstitial nephritis (active or inactive), diabetic glomerulosclerosis, thrombotic microangiopathy and focal segmental glomerulosclerosis (commonest variant - not otherwise specified) 98, 99.

Baradhi and colleagues 98 reported a case of methamphetamine-induced end-stage renal failure (ESRF) in a patient who had been taking methamphetamine for several years. It was then concluded that continuous methamphetamine use might induce persistent malignant hypertension in this patient, evidenced by high blood pressure (210/124 mm Hg) and multi-end-organ damages, such as left ventricular hypertrophy and ESRF. The renal biopsy of the patient identified multiple renal pathologies, including subacute thrombotic microangiopathic injury, advanced glomerulosclerosis, striking mucoid intimal hyperplasia, cellular crescent formation and tubular atrophy or interstitial fibrosis 98.

Malignant hypertension is a common manifestation in methamphetamine users. A retrospective study involving methamphetamine users referred to the renal unit reported that 96% of them had chronic kidney disease (55% ESRF) and 89% had hypertension (45% malignant hypertension) 97. Among patients with renal biopsy results, half showed hypertensive changes, while a quarter demonstrated malignant changes. Also, 58% of patients with mesangiocapillary glomerulonephritis had positive IgM and C3 complement 97. The vasoconstrictive effects of methamphetamine-mediated via α_1_ and β_1_ receptors may explain the reason for increased systemic vascular resistance and blood pressure 97, 98.

### Endothelial toxicity

Endothelial cells are flattened cells that form the inner cellular lining of all blood vessels 113. The cells are connected by tight junctions and anchored to a continuous basal membrane. The endothelium can be found in most arteries, veins and capillaries of the brain, skin, lungs, heart, kidneys and muscle. The cells form a natural barrier between blood and tissues, controlling the movement of substances and fluid across tissue 114. Impaired endothelial cell functions can lead to serious health issues 115. For instance, the blood-brain barrier, a highly selective membrane formed by endothelial cells using tight junctions to prevent large and potentially toxic molecules from entering the brain, can be compromised due to vascular dysfunction in certain conditions 116. One potential cause of endothelial toxicity is methamphetamine mediated through oxidative stress, inflammation and vasoconstriction.

#### Oxidative stress and inflammatory response

Oxidative stress induced by methamphetamine in brain endothelial cells can cause impaired blood-brain barrier function. Methamphetamine increases the permeability of the blood-brain barrier *in vivo*. The exposure of brain microvascular endothelial cells (BMVECs) to methamphetamine reduces the expression of cell membrane-associated tight junction proteins, resulting in a reduction in the tightness of BMVEC monolayers. Other pathological changes include increased ROS production, enhanced monocyte migration across the endothelium, altered glucose transporter protein-1 expression, reduced tight junction proteins (occludin and zonula occludens-1) and activation of myosin light chain kinase (MLCK) in BMVECs 117-119. Further, methamphetamine increases plasma levels of oxidative marker MDA, inflammatory marker C-reactive protein and endothelial injury marker endothelial-derived microparticle 120.

#### eNOS/NO-mediated transcytosis

Endothelial NOS (eNOS)/NO-mediated fluid-phase transcytosis is among the proposed mechanism for the increased permeability of methamphetamine and lymphocytes at the blood-brain barrier 121. The BMVECs respond to low concentrations of methamphetamine by a rapid activation of endothelial NOS. Inhibiting this enzyme reduces the methamphetamine-induced blood-brain barrier permeability through caveolar transport without compromising the integrity of tight junctions in preclinical models of *in vitro*, *in vivo* and *ex vivo* 121, 122. Remarkably, a similar effect is absent at higher methamphetamine concentrations 121, 122.

#### Endothelial-dependent and independent vasoconstriction

Another proposed mechanism of methamphetamine-induced vasoconstriction is the endothelin-dependent pathway. The synthesis of endothelin-1 (ET-1) by brain endothelial cells is enhanced following methamphetamine exposure. In a study using cultured mouse brain endothelial cell lines, Seo and co-workers reported that methamphetamine exposure caused vasoconstriction, which was prevented by concurrent administration of endothelin receptor antagonists 123. As previously stated, myoglobin-induced formations of potent vasoconstrictors F2-isoprostanes and ET-1 are among the mechanisms of renal toxicity induced by the use of methamphetamine use 106, 107.

Contradictory, a case-control study involving methamphetamine users and healthy controls reported no significant differences between groups in the flow-mediated dilatation (FMD), a measure of endothelium-dependent vasodilation 124. However, the researchers found a marked difference between groups in endothelium-independent vasodilation, as measured using exogenous vasodilator nitroglycerin 124. Smoker status (cigarette) in both groups might confound the effect of methamphetamine on FMD, as smoking can cause FMD impairment. Methamphetamine effects on vascular media smooth muscle may reduce the reactivity of smooth muscle cells towards nitroglycerin, causing reduced dilation in response to nitroglycerin 124. The altered reactivity of vascular smooth muscle might predispose patients to vascular diseases like stroke. A recent case-control study investigating the risk of cerebral small vessel disease in patients with acute ischemic stroke reported that patients who used methamphetamine had a higher total burden of cerebral small vessel disease and more pathological changes in white matter hyperintensities and lacunes 125.

#### Activation of apoptosis-induced pathways

Methamphetamine induces apoptosis in endothelial cells through various signalling pathways, including blockade of kappa opioid receptor, inactivation of the protein kinase B (Akt)/mammalian target of rapamycin (mTOR)/p70S6K, upregulation of the extracellular signal-regulated kinase 1/2 (ERK 1/2) 126, as well as stimulation of NADPH-Oxidase-2 (NOX-2) 127 in primary human and rat brain microvascular endothelial cells 127. Another apoptotic pathway affected by methamphetamine exposure in human umbilical veins and rat cardiac microvascular endothelial cells is Nupr1/Chop/P53/PUMA/Beclin1 128. Similarly, activation of the VEGF/PI3K/Akt/eNOS-signalling pathway by methamphetamine can also increase cardiac microvascular permeability 129. Collectively, the pathways represent potential therapeutic targets for methamphetamine-induced endothelial cell apoptosis in blood-brain barrier impairment and cardiovascular toxicity. Table 1 summarises the studies on methamphetamine-associated toxicity effects.

## Conclusion

Methamphetamine is a toxic psychostimulant with various detrimental effects on the brain, heart, liver, kidneys and eyes. Various pharmacokinetic and pharmacodynamic factors may alter the toxicity effect of methamphetamine. Understanding the mechanisms of methamphetamine toxicity is essential to develop treatment strategies to reverse or attenuate the progress of methamphetamine-associated organ damage.

## Figures and Tables

**Table 1:** Summary of human and animal studies related to ophthalmologic complications of methamphetamine use.

Author	Year	Populations/Case	Findings/Comments
Neurotoxicity			
Preclinical studies			
Su *et al.*	2020	Adult male D3R knockout (D3R-/-) mice and adult male wild-type (WT) mice. Methamphetamine was administered at a dosage of 2.0 mg/kg.	Methamphetamine caused behavioural sensitization in both D3R-/- and WT mice. ↓ Behavioural sensitization in D3R-/- mice, D1R and D2R gene expressions in NAc (in WT mice) and caudate putamen (WT and D3R-/- mice). ↑ D3R gene expression in the hippocampus, prefrontal cortex, and notably, the nucleus accumbens after the treatment.
Gibson *et al.*	2022	Adult male C57BL/6 J mice were subjected to a methamphetamine binge regimen, involving subcutaneous administration of 10 mg/kg d,l-METH, or saline at a volume of 10 mL/kg, every 2 hours, totalling four injections.	↓ Dorsomedial striatal long-term potentiation (methamphetamine or saline + SCH23390 ((dopamine D1R antagonist). ↑ Dorsomedial striatal long-term potentiation (bupropion + methamphetamine).
Da Silva Santos *et al.*	2019	Sprague Dawley rats (250-350 g) were administered four doses of methamphetamine at 4 mg/kg subcutaneously (calculated as free base), with each dose given 2 hours apart. The control group received saline.	↑ Dopamine levels in frontal cortex (at 2- and 24-hours) and amygdala (at 24 hours), ↓ 3,4-dihydroxyphenylacetic acid (DOPAC) in striatum (at 24- and 48-hours).
Miyamoto *et al.*	2014	NAc-targeted dopamine D2R knockout male C57BL/6J mice aged eight weeks old were administered methamphetamine at a dosage of 1 mg/kg via subcutaneous injection. Following methamphetamine treatment, mice treated with adeno-associated virus vectors containing a miRNA sequence for the dopamine D2R showed significantly reduced	↓ Preferred behaviours in the place conditioning test in methamphetamine-induced locomotor activity compared to non-knockout mice.
Baladi *et al.*	2014	Male Sprague-Dawley rats were administered PG01037 (4 x 32 mg/kg, subcutaneous injection) or vehicle (4 x 1 ml/kg, subcutaneous injection) at 30 min prior to methamphetamine treatment (4 x 7.5 mg/kg, subcutaneous injection, at 2-hour intervals) or vehicle (4 x 1 ml/kg, subcutaneous injection, at 2-h intervals).	↑ Body temperature (methamphetamine), striatal dopamine transporter (methamphetamine + PG01037) ↓ Striatal dopamine transporter (methamphetamine), body temperature (methamphetamine + PG01037-repeated doses)
Branch *et al.*	2012	Midbrain slices of male C57BI6J mice aged ≥ six-week-old were subjected to low (0.1-1.0 μM) and high (10 μM) concentrations of methamphetamine.	↑ Dopamine neuron activity (by activating DAT-mediated excitation) and the amplitude of inhibitory postsynaptic currents (IPSC) (low doses). ↓ Dopamine neuron activity (via activating dopamine D2 autoreceptor) and the amplitude of IPSC (high doses).
Di Monte *et al.*	1996	Male Swiss Webster mice aged 7-8 weeks received either vehicle (oil) or 7-nitroindazole (7-NI) (50 mg/kg) subcutaneously 20 minutes before each intraperitoneal injection of either methamphetamine (7.5 or 10 mg/kg) or saline administered at 2-hour intervals. The levels of striatal dopamine, DOPAC, and HVA were measured at 90 minutes, 1 or 5 days after the last methamphetamine injection.	At Day 1 (10 mg/kg) ↓ Dopamine (methamphetamine + oil vs methamphetamine + 7-NI, saline+oil, saline + 7-NI; methamphetamine + 7-NI vs saline + oil), DOPAC (methamphetamine + oil vs methamphetamine + 7-NI, saline + oil, saline + 7-NI; methamphetamine + 7-NI vs saline + oil), HVA (methamphetamine + oil vs saline + 7-NI) At Day 1 (10 mg/kg) ↓ Dopamine (methamphetamine + oil vs saline + oil, saline + 7-NI; methamphetamine + 7-NI vs methamphetamine + oil, saline + oil, saline + 7-NI), DOPAC (methamphetamine + oil vs saline + oil, saline + 7-NI; methamphetamine + 7-NI vs saline + oil, saline + 7-NI), HVA (methamphetamine + oil vs saline + oil, saline + 7-NI; methamphetamine + 7-NI vs saline + oil, saline + 7-NI)
Bowyer *et al.*	1995	Male Sprague-Dawley rats aged 4-6 months were administered four injections of 5 mg/kg methamphetamine or Ringer's solution intraperitoneally at 2-hour intervals. Nitric oxide synthase (NOS) inhibitors N^G^ -nitro-L-arginine (NOARG), N^G^ -nitro-L-arginine methyl ester (L-NAME) or D-NAME and NO generators sodium nitroprusside (SNP) and isosorbide dinitrate (ISON) were administered into the micro-dialysate of caudate/putamen with or without.	↓ Dopamine (NOARG, L-NAME, SNP, ISON), DOPAC (ISON) ↑ Dopamine (NOARG + high doses of L-arginine and L-citrulline).
Clinical studies			
Watling *et al.*	2023	A case-control study involving 14 methamphetamine users (mean age: 39.6 years) and 20 HC (mean age: 32.5 years) investigated the differences in GSH concentration in the anterior cingulate cortex and left dorsolateral prefrontal cortex using a 3T proton magnetic resonance spectroscopy (MRS).	GSH: No significant differences. Patient vs HC ↑ Eotaxin, eotaxin-3, interferon gamma-induced protein-10, MCP-1, MCP-4, MDC, MIP- α, MIP-1β, TARC, IFN-γ, IL-6, IL-8, IL-7, VEGF, MMP-1, MMP-9, MMP-10, BDNF, MPO
Su *et al.*	2020	A case-control study involving 50 patients (31.96±6.69 years) and 20 HC (29.12±6.42 years) investigated differences in neurometabolite concentrations in the left dorsolateral prefrontal cortex using a 3T MRS.	Patient vs HC ↓ GABA, GABA/Glx, PCr, GPC, Ins, NAA, GPC + PCh, Cr + PCr, NAA + NAAG ↑ GSH
Cardiotoxicity			
Preclinical studies			
Yu *et al.*	2023	Adult male C57BL/6J mice and specific-pathogen-free (SPF), Nrf2-/- knockout (Nrf2-KO) mice were randomly divided into six groups (n=6): Control, methamphetamine, sulforaphane, sulforaphane + methamphetamine, Nrf2-KO control, Nrf2-KO + methamphetamine groups. The mice received sulforaphane once daily (one hour before methamphetamine in the combined group) or methamphetamine twice daily for five days.	Methamphetamine vs control groups: ↑ Cardiac injury score, heart weight, CK-MB, LDH, cTnI, Caspase-3, Bax, MDA, Nrf2, HO-1 ↓ SOD, Bcl-2 Histological changes: Nucleolysis, nuclear fission, myocardial fibre misalignment, fibrosis (induced by methamphetamine but reversed by sulforaphane) Methamphetamine caused myocardial injury, oxidative stress, and apoptosis in Nrf2-KO mice.
Zhang *et al.*	2023	WT zebrafish aged 6-12 months were initially treated with methamphetamine three times a week over a 2-week duration with a pre-treatment open chest surgery to improve subsequent ECG signal acquisition. cAMP expression and Ca2+ regulation in cardiomyocytes were measured.	Methamphetamine-treated vs untreated zebrafish: ↑ Collagen type I, cAMP, calcium, lysyl oxidase, lysyl hydroxylase, heart rate variation ↓ QTc interval, PR interval (week 1) ↓↑ Heart rate (peaked at week 1 and decreased in week 2) Summary: Methamphetamine induced fibrosis and arrhythmia secondary to downstream effectors of cAMP.
Chavva & Rorabaugh	2022	The pregnant female Sprague-Dawley rats were divided into four experimental groups - control, methamphetamine, saline (Day 1-11) + methamphetamine (Day 12-22), and methamphetamine (Day 1-11) + saline (Day 12-22) subcutaneously. An *ex vivo* experiment was conducted in adult female rats where an ischemic-perfusion condition was performed.	↑ Infarct size (methamphetamine exposure in the first- or second half of the pregnancy). NS: HR, developed pressure, +dP/dT, -dP/dT, coronary flow rate
Kolluru *et al.*	2022	Male WT C57BL6/J mice aged 12 weeks were randomly assigned to different experimental groups and - Na_2_S drinking solution containing methamphetamine or saline. In experiment two (binge and crash), C57BL/6J male mice received 0-6 mg/kg methamphetamine subcutaneously or saline five days a week for four weeks.	Methamphetamine-treated mice vs control: Skeletal muscle: ↑ Superoxide, dihydroethidium (DHE), NOX2 subunit gp91^phox^ and p47^phox^, ICAM-1, VCAM-1, fibrosis area ↓ Cystathionine γ-lyase (CSE), total sulfide, total Nox, p-eNOS ATF4, SIRT1, SIRT4 Heart: ↑ NOX2 subunit gp91^phox^ and p47^phox^ ↓ EF, FS, CSE, ATF4, SIRT1, SIRT4 Plasma/blood: ↑ Superoxide ↓ CSE, H_2_S (acid-labile, bound sulfane sulfur, total sulfide), NO (free nitrite, S-nitroso thiol, total NO), flow-mediated vasodilation and blood flow velocity Exogenous sulfide therapy or endothelial cell CSE transgenic overexpression (treatment + methamphetamine vs methamphetamine only) ↑ Flow-mediated vasodilation, blood flow velocity, CSE activity, total Nox (plasma, muscle), total sulfide (muscle) ↓ gp91^phox^, p47^phox^, ICAM-1, VCAM-1 Summary: Methamphetamine affected cardiovascular function via a CSE/H_2_S/NO-dependent pathway. NOX2 activation has profound profibrotic effects.
Dague *et al.*	2022	Pregnant Sprague-Dawley rats received either saline or methamphetamine (5 mg/kg/day) once per day subcutaneously starting on day 1 gestation until the pups were born. The pups were weaned at postnatal Day 28 and sacrificed at week 8.	Female vs male offspring ↑ Cardiac gene expression changes in female ↓ Dimethylarginine dimethylaminohydrolase-2 (female only) and 3-hydroxybutyrate dehydrogenase 1 (both sexes) Summary: Prenatal methamphetamine exposure promotes sex-specific altered cardiac gene expression that persisted in adult rats.
Chavva *et al.*	2021	Forty-eight adult male and female Sprague Dawley rats aged eight weeks were divided into eight experimental groups (n=6/group): males on either saline or methamphetamine (5 mg/ kg/day) subcutaneously for ten- days, females on either saline or methamphetamine for ten days, males/females on either saline/methamphetamine for ten days followed by 30 days of abstinence.	↑ Cardiac gene expression changes in female rats were primarily related to circadian clock regulatory genes (*Dbp, Per3, Per2, Bmal1,* and* Npas2*) Changes returned to baseline values following 30 days of abstinence. Summary: Methamphetamine-induced cardiac gene expression changes were more prominent in female rats and reversible following abstinence.
Abdullah *et al.*	2020	Male C57BL/6 mice aged 8-10 weeks were randomized to receive saline or methamphetamine (0-6 mg/kg) subcutaneously, four injections /day (two hours apart), five days/week for four weeks.	Methamphetamine-treated vs vehicle-treated mice ↓ FS, EF, heart size, heart weight-to-tibia length, *Myh6*, mitochondrial respiration, mitochondrial fission 1 protein (Fis1) and Sigmar1 protein expression ↑ LV internal dimension and LV volume at systole, fetal cardiac gene expression (*Nppa, Nppb, Myh7*), fibrosis area, heart collagen deposition. (+) periostin, and α smooth muscle actin Summary: Methamphetamine induced mitochondrial dysfunction, cardiac remodelling and hypertrophy leading to contractile dysfunction. Sigmar1 is a therapeutic target for methamphetamine-associated cardiomyopathy.
Freeling & McFadden	2020	Adult Sprague Dawley male rats received either self-administered methamphetamine (8 h/d) or saline for nine days. The jugular catheter was inserted to allow methamphetamine (0.12 mg/10 uL) or saline infusion, followed by a 30-second timeout period whenever a rat pressed the active lever (maximum nine presses), which also resulted in the delivery of 45 mg food pellets. On the 4^th^ and 9^th^ self-administration sessions, an echocardiogram was performed following a low dose of methamphetamine (1 mg/kg).	Methamphetamine vs saline Overall ↑ Lever presses, methamphetamine intake (methamphetamine) ↓ Weight gain Following four days NS: SV, HR, EF, diastolic LV posterior wall thickness Following nine days ↑ HR, EF ↓ SV, diastolic LV posterior wall thickness Summary: Methamphetamine causes structural and functional changes of the heart.
Marcinko *et al.*	2019	C57BL/6 mice aged 8-10-weeks were administered a gradual increase of methamphetamine dose over two study periods: 2- (4-5 mg/week increment for eight weeks up to 35 mg/kg) and 5- month (2 mg/week for 20 weeks up to 40 mg/kg). Control mice received PBS.	At two months Female (methamphetamine vs control) NS: EF, FS, LVID(d) and LVID(s) Male (methamphetamine vs control) ↑ LVID(s) ↓ FS At five months Female (methamphetamine vs control) ↓ Fibrosis Male (methamphetamine vs control) ↑ LVID(s), LVID(d), fibrosis, mortality ↓ EF and FS
Rorabaugh *et al.*	2017	Sprague Dawley male and female rats aged eight weeks were injected methamphetamine (5 mg/ kg/day) or saline once daily (subcutaneously) for ten consecutive days and then subjected to a 20-minute ischemic period.	Female (methamphetamine vs control) after the ischemic insult ↑ Infarct size, end diastolic pressure ↓ +dP/dT, -dP/dT, developed pressure Persisted hypersensitivity to ischemic injury in female hearts following one-month methamphetamine abstinence. Males (methamphetamine vs control) NS Summary: Exposure of adult rats to methamphetamine is sex-dependent in favour of female rats in increasing the extent of myocardial injury, following an ischemic insult.
Vaupel *et al.*	2016	Age-matched rhesus macaques (*Macaca mulatta*) were compared between groups; a self-administered methamphetamine group (9.5 ± 0.8 year, 8 males, n=8) vs control group (10.6 ± 0.2 year, 9 males and 1 female, n=10). Methamphetamine exposure: 5.5 ± 0.7 year. Methamphetamine challenge (0.35 mg/kg) at minute 10.	Methamphetamine vs control ↑ HR, BP ↓ LVEF, CO at three months Summary: Persisted methamphetamine cardiovascular effects even after prolonged abstinence which may underlie clinically reported acute cardiotoxic events.
Clinical studies			
Abdullah *et al.*	2023	Thirty-four methamphetamine-positive and seven control autopsy samples.	Methamphetamine (+) vs control ↑ Collagen (perivascular and interstitial spaces of the myocardium), fibrosis, heart weight.
Batra *et al.*	2022	Seven hundred eighty-three CVD patients only (control) and 593 cardiovascular patients with a history of methamphetamine use.	Methamphetamine vs control: ↑ 12-fold in subjects who showed a premature onset of CVD (*<*30 years of age). ↓ First CVD diagnosis (± 8 years, earlier in blacks than whites). Hypertension was the most prevalent CVD, followed by congestive heart failure, coronary artery disease, and hyperlipidemia.
Curran *et al.*	2022	66 199 out of 20 249 026 patients used methamphetamine (63% males).	Development of CVD subtypes: Heart failure (HRa: 1.53, 95%CI: 1.45-1.62) Pulmonary hypertension (HRa: 1.42, 95%CI: 1.26-1.60) Myocardial infarction (HRa: 1.19, 95%CI: 1.08-1.31) Risk factors: Chronic kidney disease (HRa: 2.38, 95%CI: 1.74-3.25) Hypertension (HRa: 2.26, 95%CI: 2.03-2.51) Diabetes (HRa: 1.75, 95%CI: 1.55-1.97) Smoking (HRa: 1.28, 95%CI: 1.17-1.40)
Stokes *et al.*	2021	Thirty patients (43.7 ± 7.5 years, 26 males) with methamphetamine-associated cardiomyopathy (13.4+5 years of methamphetamine use; 53% smoked, 43% of IV users)	Severe LV and moderate-to-severe RV dilatations (volumes) and dysfunctions (EF). 73.3% of patients had myocardial late gadolinium enhancement (LGE) - Interventricular septum/mid-wall (59.1%), sub-endocardial (22.7%), and transmural regions (18.2%) 20% of patients had intracardiac thrombus.
Bhatia *et al.*	2021	A retrospective study involving methamphetamine-associated heart failure patients with reduced left ventricular ejection fraction (reduced EF) (n=28), methamphetamine-associated heart failure patients with preserved left ventricular ejection fraction (preserved EF)(n=28) and heart failure controls without methamphetamine use (n=23 (reduced EF), 31 (preserved EF)).	Effect of cessation of methamphetamine in reduced EF patients vs continued use ↑ EF ↓ Heart failure admissions Methamphetamine patients with preserved EF vs controls: ↑ Baseline TR velocity, and right ventricular systolic pressure ↓ Lateral E/E' ratio Cessation of methamphetamine did not cause any significant improvement in echocardiographic parameters in the preserved EF group.
Jariwal *et al.*	2021	A retrospective case-control study involving 254 patients with methamphetamine-associated cardiomyopathy and 268 patients without methamphetamine-associated cardiomyopathy.	Methamphetamine vs control ↓ (<) LVEF ↑ (>) LV mass index, LVEDV index
Zheng *et al.*	2019	A case-control study involving 22 male methamphetamine abusers (11 without chest pain, 11 with chest pain), free of CAD and 22 age-matched male healthy participants.	Methamphetamine users vs healthy controls: ↑ (>) BP, LV mass index, impaired diastolic function Methamphetamine users with chest pain vs without chest pain and healthy: ↑ HR, arrival time ↓ (<) Peak intensity (functional capillary volumes), ascending slopes (microvascular flow velocities) Methamphetamine users with chest pain vs healthy controls ↑ (>) Contrast agent arrival times ↓ (<) Functional capillary blood volumes, microvascular flow velocities, myocardial perfusion.
Akhgari *et al.*	2017	One hundred methamphetamine poisoning-related deaths of young adults aged 21-35 years compared to 100 control cases.	68% cardiovascular pathology. Histopathologic features: myocardial fibre hypertrophy, myocardial infarction, interstitial and perivascular fibrosis, atherosclerosis and focal degeneration/necrosis.
Hepatotoxicity			
Preclinical studies			
Soo *et al.*	2020	Female guinea pigs (n=4/group) were divided into control and methamphetamine groups (subcutaneously at a dosage of 5 mg/kg for three alternate days).	↓ CYP1A2 mRNA expression, 11β-hydroxysteroid dehydrogenase-1 mRNA expression. ↑ CYP1A2 enzyme activity. Comments: Methamphetamine administration has the potential to alter drug metabolism via the CYP1A2 metabolic pathway.
Azizi *et al.*	2023	Rats (n=5/group) administered methamphetamine subcutaneously at a dosage of 0.5 mg/kg for 21 days.	Microscopic vacuolar degeneration and congestion were observed in both methamphetamine and control groups without a significant difference.
Wang *et al.*	2022	WT mice (n=10/group) were administered IV methamphetamine at doses of 1.5, 4.5, and 7.5 mg/kg once a day for two days per dose, and then 8 injections were administered at a dose of 10.0 mg/kg four times per day at 2 h intervals.	H&E staining revealed shrinkage of the nuclei, loose cytoplasm, and extensive vacuolar degeneration of hepatocytes. ↑ Serum levels of AST and ALT, mRNAs and proteins of TLR4-mediated proinflammatory cytokines. Comments: TRP channels, TLR-signalling pathway, NF-κB signalling pathway and TNF signalling pathway were activated, leading to an increased inflammatory response and hepatotoxicity.
Zhang *et al.*	2022	C57BL male mice (n=6/group) were injected with 15 mg/kg methamphetamine via the IP route (treatment group).	Weight loss, cholestasis, hepatic dysfunction, histopathological changes. ↑ Liver/body weight, hepatic glycogen, ALT, AST, ALP, and LDH indexes, protein levels of TLR4, MyD88, and NF- κB, mRNA levels of TNF- α, IL-6, IL-1β, IL-18, modulatory effects on caecal microbiota. ↓ Body weight Comments: Stimulatory effects on inflammation via TLR4/mMyD88/NF-κB signalling pathway and caecal microbiota modulatory effects.
Chen *et al.*	2021a	BABL/c mice (n= 8/group). The treatment group received an increasing dosage of methamphetamine IP as follows: days 1-2, 1.5 mg/kg; days 3-4, 4.5 mg/kg; days 5-6, 7.5 mg/kg; and days 7-8, 10 mg/kg, four injections a day every 2 h.	Methamphetamine caused: ↑ ALT, AST, ROS, SOD, TLR4, MyD88, TRAF6 (antibiotic treatment alleviated the methamphetamine effects). ↓ Body weight, liver weight. Presence of karyopyknosis and extensive cytoplasmic damage (vacuolar degeneration). Comments: Propose relationship between methamphetamine-induced hepatotoxicity through oxidative stress TLR4/MyD88/TRAF6 pathway regulated by gut microbiota.
Xie *et al.*	2018	Five-week-old of Sprague Dawley rats (n= 6/group; Total=18). The treatment groups received 8 IP injections of METH (15 mg/ml/kg body weight/injection) at 12 h intervals.	Methamphetamine caused: Extensive cytoplasmic damage (vacuolar degeneration) (lactulose pre-treatment reversed the abnormality).
Halpin *et al.*	2012	Male Sprague Dawley rats were treated with methamphetamine 10 mg/kg intraperitoneally every 2 h or saline.	↑ Serum levels of AST, ALT, and ammonia (lactulose treatment prevented the increase in ammonia levels. Vascular and sinusoidal congestion and cytoplasmic damage.
Koriem & Soliman	2014	Albino rats (n= 8 /group). The group receiving methamphetamine were injected with 10 mg/kg IP twice a day for seven days.	↑ Serum levels of AST, ALT, ALP, bilirubin, cholesterol, LDL, TG, and MDA (also in the liver and brain), and plasma levels of NO (also in the liver). ↓ Serum levels of total protein, albumin, globulin, and albumin/globulin ratio, and blood and liver levels of SOD and GPx. A band of oedema in the periportal area compressing the surrounding hepatocyte, cytoplasmic vacuolation, and polysaccharides inclusions. Treatment with chlorogenic and caftaric acid reversed the abnormalities caused by methamphetamine. Comments: Methamphetamine causes oxidative stress and hepatotoxicity and polyphenols have a protective effect against it.
Wang *et al.*	2017	Adult male Sprague Dawley rats (6 weeks old, n=6/group). The methamphetamine group received IP injections of 15 mg/ml/kg body weight/injection, at 12 h intervals for eight times).	↑ Serum levels of ammonia, AST and ALT. Presence of extensive cytoplasmic damage (vacuolar degeneration).
Chen *et al.*	2023	Male BALB/c mice (n=8/group). The methamphetamine group received an increasing dose of methamphetamine IP as follows: days 1-2, 1.5 mg/kg once daily; days 3-4, 4.5 mg/kg once daily; days 5-6, 7.5 mg/kg once daily; and days 7-8, 10 mg/kg, four injections a day every two hours.	↓ Body weight, liver weight, ↑ ALT, AST, ALP, protein levels of TLR4, MyD88, NF-κB, IL-1β, and IL-6. Hypertrophic hepatocytes, disarray, loss of the cytoplasm, glycogen storage, migration of their nuclei, acidophil bodies, and infiltration of inflammatory cells in portal and/or lobular areas. Comments: Induction of methamphetamine hepatotoxicity by inflammation via TLR4/MyD88/NF-κB signalling pathway activation.
Clinical studies			
Karch *et al.*	1999	A case-control study involving 527 autopsy samples of 413 methamphetamine-related death and non-methamphetamine-related death.	Cirrhosis and fatty liver were common in both cases and controls. Hepatitis and triaditis were significantly higher in intravenous drug users regardless of drug type (including methamphetamine).
Merchant *et al.*	2019	An autopsy report of a 35-year-old man with methamphetamine overdose that caused death due to acute pancreatitis and acute hepatic failure.	Gross examination revealed hepatic steatosis, cystic, and necrosis areas, and necrotic bile duct hamartomas. Microscopic examination reported necrotic hamartoma, extensive fibrosis, and hepatic thrombi. The histopathological findings of pancreatic tissue revealed multiple areas of tissue and fat necrosis with infiltration of acute inflammatory cells, edema and fibrinous exudate.
Ma *et al.*	2022	First cohort - Thirty males with methamphetamine use disorder (duration: 61-107 months) undergoing withdrawal and 30 male HC aged 25-50 years. Major routes of methamphetamine intake were smoking or nasal inhalation. Second cohort - 10 HC with 10 patients with methamphetamine use disorder in each group; 7-day, 3-month and 12-month withdrawal stages.	↑ Patients > HC: AST, AST, depressive and anxiety symptoms/scores. TG, HDL, LDL, and total bilirubin, and LCA:CDCA ratio. ↓ Patients < HC: Total bile acid (BA), d4-cholic acid (CA), and chenodeoxycholic acid (CDCA) with a maximum reduction at three months. ↓ Hyocholic acid (HCA) and ursodeoxycholic acid (UDCA) levels at 3 months. ↓ CA/CDCA and deoxycholic acid (DCA)/CA. Significant correlations: HAM-A (anxiety score) ↔ ALT, AST, and LDL, HAM-D (depression score) ↔ AST, TG, HDL, and AST/ALT ratio, TLCA/CDCA ratio ↔ ALT and AST, LCA/CDCA ↔ ALT and AST, Bas ↔ HAM-A (negative correlation).
Kamijo *et al.*	2002	A case of a 41-year-old Pakistani man who presented with psychosis, hyperthermia, rhabdomyolysis, and liver dysfunction after intravenous injection of methamphetamine.	A liver biopsy showed confluent necrosis and ballooning degeneration in centrilobular zones.
Ocular toxicity			
Preclinical studies			
Lee *et al.*	2021	C57BL/6 male mice aged eight weeks received either bacteriostatic saline (body weight of 28.8 ± 0.6 g) or increasing methamphetamine (29.1 ± 0.8 g) dose (0-6 mg/kg) four times daily with two hours intervals over 4 weeks.	↑ Vessel length density in the mid-peripheral and peripheral retina (D26), number of arterioles (D26), VEGFa (D12&D26), pimonidazole (D12&D26), HIF-1α (D12&D26).
Lee *et al.*	2020	C57BL/6 male mice aged eight weeks received either bacteriostatic saline (body weight of 27.7 ± 1.8 g) or increasing methamphetamine (28.1 ± 1.5 g) dose (0-6 mg/kg) four times daily over five days.	↓ Thickness of the whole retina, outer nuclear layer, outer plexiform layer, inner nuclear layer, inner plexiform layer, retinal nuclei number (ONL and INL), PECAM-1 (CRA), glypican-1 (CRA), syndecan-1 (CRA), ↑ Norepinephrine (plasma levels), TUNEL-positive cells (RPE, ONL, INL, GCL), TNF-α (retina & blood vessels), GFAP, MMP 2 & 9 (plasma & CRA), MMP-14 (retina & blood vessels).
Melo *et al.*	2010	Adult male Wistar rats at postnatal day 91 were treated with either saline or methamphetamine (5 mg/kg) IP four times every two hours daily with over 10 days.	In both retina and plasma ↓ Total antioxidant status ↑ MDA, SOD, NO
Lai *et al.*	2009	CD1 mice aged eight weeks with weights of 23-25 g were treated with 40 mg/kg single dose IP (Group 1), 10 mg/kg IP four times with two-hour intervals for one day (Group 2) and 40 mg/kg normal saline (Group 3) (six mice per group).	↑ GFAP in IPL (Group 1), S-100 in IPL & INL (Group 1), CD11b staining (Group 1)
Clinical studies			
Tang & Chong	2022	A case report of a 57-year-old man with left eye vision loss and no comorbidities. He had a history of methamphetamine use (smoking).	The left eye vision was finger count. On fundoscopy, both eyes had flame haemorrhages and cotton wool spots, but the left was more widespread.
Talebnejad *et al.*	2020	A case-control study involving 55 methamphetamine users and 49 HCs aged 44.6±1.0 and 43.1 ± 0.9, respectively (all males). The mean duration and dose of methamphetamine use were 4.5 ± 4.0 years and 0.2 ± 0.2 g, respectively. The majority (74.5%) consumed the drug by inhalation (smoking).	↓ Retinal nerve fibre layer thickness (RFNL) & Bruch's membrane opening minimum rim width (MRW). NS: ganglion cell layer thickness - between daily dose and RFNL thickness & MRW.
Guo *et al.*	2019	A 37-year-old male with a history of chronic methamphetamine use via intranasal route for seven years. The right eye vision (20/50 to 20/25) and the left eye vitreous haemorrhage improved after pan-retinal photocoagulation. However, the left eye vision (20/200) remained unchanged.	Ischemic retinopathy.
Kumar *et al.*	2006	A case report of a 48-year-old man with Graves ophthalmopathy on methimazole, prednisolone and artificial tears. He is a smoker (cigarettes and marijuana) and regularly snorting methamphetamine (several days a week for years).	The visual acuities were 20/25 OD and 20/20 OS. Crystalline talc deposits in the macula of both eyes were observed on fundoscopy.
Wijaya *et al.*	1999	A 35-year-old male with underlying diabetes mellitus presented with irreversible visual loss three days after intranasal methamphetamine use. The characteristics of the clinical presentation, including pale atrophic optic nerve head, had similarities with the classical non-arteritic ischemic optic neuropathy.	Ischemic optic neuropathy.
Poulsen *et al.*	1996	Four keratitis cases of three female patients aged 28-42 years and a 56-year-old male patient. All patients abuse methamphetamine via inhaled routes. However, only two exclusively inhaled, while the other two also administered methamphetamine via IV route and one smoked. One patient had underlying systemic lupus erythematous.	Various microorganisms were isolated from the sensitivity tests, including Streptococcus viridans, Group D Streptococcus, Staphylococcus aureus, Propionibacterium acnes, Pseudomonas, Capnocytophaga, and Candida albicans.
Chuck *et al.*	1996	A case study of a 31-year-old female patient with recurrent keratitis (six times) which was often preceded by heavy methamphetamine use via inhalation. She also reported multiple history of trauma to the eyes.	Streptococcus viridans was the only microorganism isolated during the keratitis episodes. The visual acuity changed from 20/200 OD and 20/30 OS (1993) to 20/40 OD and 20/70 OS (1995).
Wallace et el.	1992	A case report of a 26-year-old woman without underlying comorbidities who snorted methamphetamine a few hours before the presentation of blurred vision and a spot in her left eye.	On fundoscopy, multiple intra-retinal haemorrhages and dilated veins were reported.
Shaw *et al.*	1985	A case report of a 26-year-old man who developed amaurosis fugax and then retinal vasculitis (right eye). He had a history of polysubstance abuse (marijuana, alcohol, cocaine and methamphetamine). The most recent drug use was methamphetamine intranasally (right nostril).	The authors postulated methamphetamine-induced vasospasm as the cause of amaurosis fugax, while methamphetamine-induced hypersensitivity was the cause of retinal vasculitis.
Renal toxicities			
Preclinical studies			
Azizi *et al.*	2023	Rats (n=5/group) administered methamphetamine subcutaneously at a dosage of 0.5 mg/kg for 21 days.	Methamphetamine-receiving rats: Atrophied glomeruli, dilated urinary space of Bowman's capsule, areas of necrosis in proximal and distal tubules, eosinophilic substances in some tubules, and infiltration of mononuclear cells (particularly lymphocytes in the interstitial tissues).
Dong *et al.*	2023	Adult male C57BL/6J mice (18-20g). WT mice were randomly grouped into four groups: control (saline IP twice daily for five days), methamphetamine group (2 mg/kg twice daily for five days), sulforaphane (SFN) (10 mg/kg once a day), and sulforaphane (SFN + methamphetamine) (10 mg/kg once a day) + followed by one hour before the administration of methamphetamine.	Compared to methamphetamine-only group ↓ Creatinine, urea, kidney injury molecule-1, proximal tubule damage, MDA, Beclin 1, LC3, Nrf2, HO-1 in control and SFN + methamphetamine groups ↑ SOD (control) and Nrf2, HO-1 (SFN + methamphetamine)
Koriem *et al.*	2013	Thirty-two male Sprague Dawley rats (140-150 g) were divided into four groups: Group 1 (saline 1 mL/kg IP twice daily for five days), Group 2 (methamphetamine 10 mg/kg IP twice daily for five days), Group 3 (caffeic acid 100 mg/kg IP one day before methamphetamine injections (10 mg/kg IP twice daily for five days)), and Group 4 (caffeic acid 200 mg/kg IP one day before methamphetamine injections (10 mg/kg IP twice daily for five days)).	Compared to methamphetamine-only group ↑ GSH, GPx (control, caffeic acid only, caffeic acid + methamphetamine in kidneys, livers, and brains) ↓ MDA, protein carbonyls, catalase (control, NACA only, NACA + methamphetamine in kidneys, liver, and brain)
Zhang *et al.*	2012	Male CD-1 mice (seven weeks) were divided into four groups: control (saline), methamphetamine only, *N*-acetylcysteine amide (NACA) only, and methamphetamine + NACA (250 mg/kg). The dose for methamphetamine was 10 mg/kg every two hours (total four injections). Either saline (control, methamphetamine only) or NACA (NACA only, NACA + methamphetamine) was injected intraperitoneally 30 minutes before methamphetamine injections.	Compared to methamphetamine-only group ↑ GSH, GPx (control, NACA only, NACA + methamphetamine in kidneys, livers, and brains) ↓ MDA, protein carbonyls, catalase (control, NACA only, NACA + methamphetamine in kidneys, livers, and brains)
Clinical studies			
Choung *et al.*	2024	One-hundred-and-twelve renal biopsies of patients with a significant history of methamphetamine use (62 patients with methamphetamine use only and 50 patients with methamphetamine + other drug use were examined for any renal pathologies.	Methamphetamine use only: 97% Kidney dysfunction 65% Proteinuria 27% Haematuria Methamphetamine use only vs methamphetamine plus other substances Acute tubular necrosis 66% (19% had myoglobin casts) vs 72% (25% had myoglobin casts) Focal segmental glomerulosclerosis (FSGS) 53% (common variants: 76% not otherwise specified, 18% collapsing FSGS) vs 42% (81 % not otherwise specified, 10% collapsing FSGS) Tubulointerstitial nephritis 37% vs 36% Thrombotic microangiopathy 24% vs 32% Diabetic glomerulosclerosis 31% vs 10% Glomerulonephritis (GN) Infection-related GN 15% vs 14% IgAN 11% vs 12 %
Chansaengpetch *et al.*	2023	A 46-year-old man was found dead in custody for a minor offence.	Findings: Multiple minor contusions and abrasions, fractured three lower left ribs, dark reddish-brown urine, lethal levels of methamphetamine and amphetamine, elevated blood myoglobin, creatine kinase and creatinine levels, and diffuse tubular injury with intraluminal granular myoglobin casts.
Isoardi *et al.*	2020	A prospective observational study involving 50 presentations of AKI at the Emergency Department among patients with methamphetamine intoxication.	90% AKI 44% Rhabdomyolysis 36% ↑ Cystatin C 10% ↑ Neutrophil gelatinase-associated lipocalin
Gurel	2016	A 32-year-old man presented with muscle weakness, pain, and oliguria with a history of oral methamphetamine intake one week before admission.	Clinical findings: Acidotic breathing, skin paleness and bruises in the lumbar region, lumbosacral radiculopathy. ↑ Creatine kinase, LDH, urea, AST, ALT, potassium The patient required specialized care for his condition, including five rounds of haemodialysis due to persistent uraemia and hyperkalaemia. Subsequently, the patient improved after 12 days of hospitalization.
Baradhi *et al.*	2019	A 26-year-old Caucasian man presented with fatigue, shortness of breath, uncontrolled hypertension, epistaxis, and easily bruised. He had been taking crystal meth on a weekly basis for several years.	Clinical presentations: Tachycardia, high blood pressure (210/124 mm Hg), skin ecchymosis over thighs, deranged renal profile, metabolic acidosis, proteinuria, haematuria, anaemia, thrombocytopaenia, increased creatinine kinase, increased LDH, left ventricular hypertrophy (with EF of 45%), normal haptoglobin, normal bilirubin, negative HIV and hepatitis. Renal biopsy: Subacute thrombotic microangiopathic injury, advanced glomerulosclerosis, striking mucoid intimal hyperplasia, cellular crescent formation, and tubular atrophy or interstitial fibrosis.
Jones & Rayner	2015	Forty-seven patients with a history of methamphetamine use (85% males, aged 29 ± 8 years) referred to the renal unit were included in the study	89% had hypertension (45% classified as malignant). 72% had target organ damage (including left ventricular hypertrophy, grade III-IV hypertensive retinopathy, fibrinoid necrosis and onion skinning of the arterioles associated with crenation of the glomerular basement membrane). 96% had chronic kidney disease (CKD) (55% had CKD stage V).
Ago *et al.*	2006	A first-time user Japanese man in his late twenties immediately collapsed after he received an intravenous methamphetamine (20 mg). He was in a deep coma state for nine days before he died. The cause of death made by the physician was methamphetamine intoxication, with a direct cause of acute renal failure.	Clinical findings: Hypertension, tachycardia, pyrexia, positive urine for methamphetamine, abnormal electrocardiogram (ST depression and a negative T wave), severe brain oedema, bilateral lung infiltrative shadows, and elevated creatine kinase and potassium levels. Autopsy findings: Severe brain oedema and subarachnoid haemorrhages, myocardial necrosis, bronchopneumonia, rhabdomyolysis, deranged renal profile (↑ urea, creatinine), positive renal pathology (interstitial oedema in the renal medulla and myoglobin granular casts in the distal tubules), and liver congestion.
Ishigami *et al.*	2003	An immunohistochemical study of the kidney was conducted in a sample of twenty-two autopsy cases with positive blood (and/or urine) methamphetamine.	Methamphetamine concentration: ↑ Myoglobin-positive > myoglobin-negative 77% Myoglobin-positive 80% Myoglobin-positive in heat shock protein-70 cases 60% Myoglobin-positive in 8-hydroxy-2' -deoxyguanosine cases 82% Myoglobin-positive in 4-hydroxy-2 -nonenal cases 78% Myoglobin-positive in Cu/Zn superoxide dismutase cases Nil: *c-fos* and TNF-α
Endothelial toxicity			
Preclinical studies			
Chang *et al.*	2022	An *ex vivo* model utilising the brain of adult female Wistar rats. Both common carotid arteries were perfused with Evans Blue-albumin (EB-Alb) dye and methamphetamine (1 μM) or saline. Double carotid perfused brains were treated with or without methamphetamine and leakage tracer (horseradish peroxidase). The brain microvessels were analysed by diaminobenzidine (DAB) electron microscope.	A strong and widespread accumulation of EB-Alb dye outside the vasculature in the methamphetamine-administered hemisphere, suggesting leakage and BBB breakdown. Paracellular junctions: Intact tight junctions. Microvascular endothelial cells in methamphetamine-treated hemispheres: a large DAB + number of caveolae-containing DA vesicles.
Chen *et al.*	2022	Adult male C57 BL/6 mice were divided randomly into 3 groups: control group (saline), methamphetamine group (Two 10 mg/kg IP injections twice daily for 4 weeks), and bevacizumab + methamphetamine group (methamphetamine + bevacizumab 5 mg/kg IV twice a week for 4 weeks). Human umbilical vein endothelial cells (HUVECs): Dose-dependent: Methamphetamine concentrations of 0, 0.75, 1.0, 1.25, 1.5 and 2.0 mM for 24 h Time-dependent: 1.25 mM methamphetamine for 0, 2, 6, 12, 24, and 36 h. Then the cells were collected for western blot analysis. Rat cardiac microvascular endothelial cells (CMECs): Dose-dependent: Methamphetamine concentrations of 0, 0.2, 0.4, 0.6, 0.8, and 1.0 mM for 24 hours Time-dependent: 0.4 mM methamphetamine for 0, 2, 6, 12, 24, and 36 h. Then the cells were collected for western blot analysis.	Methamphetamine (1.50 mM for 24 h) on HUVECs and CMECs compared to control: ↑ Expressions of VEGF, PI3K, phosphor Ser473 Akt and p-eNOS compared to the control group. Methamphetamine (1.50 mM for 24 h) on HUVECs and CMECs compared to methamphetamine + Bevacizumab group: ↑ Expressions of VEGF, PI3K, phosphor Ser473 Akt and p-eNOS compared to the control group.
Hwang *et al.*	2020	Primary human brain microvascular endothelial cells (BMVECs) were treated with 1 mM of methamphetamine for 30 min and 60 min.	Methamphetamine ↑ ROS generation (30 and 60 min).
Nazari *et al.*	2018	Male Wistar rats were randomly divided into seven groups, including control, saline-1, saline-7, saline-14 and methamphetamine-1, methamphetamine-7 and methamphetamine-14 groups. Methamphetamine was administered intraperitoneally at a dose of 4 mg/kg over 1, 7, and 14 days. Plasma levels of MDA, C-reactive proteins, and endothelial-derived microparticles (using markers of Annexin V, CD144, CD31, and CD 41a antigens) were assessed.	Methamphetamine compared to the control group: ↑ Plasma levels (methamphetamine-7 and methamphetamine-14) of Annexin V + CD144 + CD41 + CD31 + MP, C-reactive protein, and MDA
Cai *et al.*	2016	*In vitro* study HUVECs and rat CMECs were exposed to saline vehicle or methamphetamine (1.25 mM for HUVECs and 0.5 mM for CMECs) for 24 h. *In vivo* study Adult male Sprague-Dawley rats received either IP injections of saline or methamphetamine (8 injections, 15 mg/kg/injection, at 12 h intervals).	Methamphetamine (compared to control) in HUVECs and CMECs (*in vivo* and *in vitro*) ↑ Nupr1, cleaved caspase-3, cleaved PARP, Bax, Bax/Bcl-2 ratio, cyto c protein (cytoplasmic fraction), Chop, ↓ Bcl-2, cyto c protein (mitochondrial fraction) Nupr1 knockdown (compared to the WT methamphetamine group) ↓ Bax, Bax/Bcl-2 ratio, cyto c protein (cytoplasmic fraction) ↑ Bcl-2, cyto c protein (mitochondrial fraction)
Jumnongprakhon *et al.*	2016	Primary rat BMVECs were isolated from neonatal rats and exposed to methamphetamine at 100 μM for 24 hours.	Methamphetamine (compared to control) ↑ ROS, reactive nitrogen species, paracellular permeability ↓ tight junction proteins (claudin-5, ccludin, zonula occludens-1) Melatonin ↓ methamphetamine-impaired tight junction via NOX-2.
Seo *et al.*	2016	Mouse brain endothelial cell lines (bEnd.3) were exposed to methamphetamine (100 μM and 1 MM) before the measurement of ET-1 levels.	Methamphetamine (compared to control) ↑ ET-1 (at 60 minutes, 1 mM), NOS3 (24 hours, 1 mM), vasoconstriction (0.1 and 1 mM in rat's cerebral arterioles).
Ma *et al.*	2014	Primary human brain microvascular endothelial cells (HBMECs) and HUVECs were treated with METH 1 μM for 24 and 48 hours.	Methamphetamine ↑ Beclin 1 and LC3, ERK 1/2 pathway ↓ Akt/mTOR/p70S6K pathway Concurrent blockade of the kappa opioid receptor and autophagic inhibitor treatment accelerated methamphetamine-induced apoptosis.
Martins *et al.*	2013	Primary BMVECs were either left untreated or treated with 1 or 50 µM methamphetamine.	Methamphetamine Unchanged: VE-cadherin, occludin, claudin-5 or ZO-1 staining. ↑ Time and concentration-dependent vesicular uptake of horseradish peroxidase
Abdul Muneer *et al.*	2011	Primary human brain endothelial cells (HBECs) were then treated with 20 μM and 200 μM methamphetamine for 24 hours.	Low concentration of methamphetamine (20 μM) ↑ Glucose transporter protein-1 (GLUT1) protein levels NS: Glucose uptake. High concentration of methamphetamine (200 μM) ↓ Glucose uptake and GLUT1 protein levels, tight junction protein (occludin and ZO-1)
Ramirez *et al.*	2009	Four-week-old male NOD/CB-17 SCID mice received seven subcutaneous injections of 1.5-10.0 mg/kg methamphetamine or sterile saline. BMVECs were cultured *in vitro* from resected epileptogenic cortex tissue obtained during surgery.	Methamphetamine: ↑ BBB permeability, ROS, gap formation. ↓ Occludin, claudin-5
Clinical studies			
Zu *et al.*	2023	Sixty-one methamphetamine users and 1297 controls were compared to determine the risk of cerebral small vessel disease (cSVD).	Methamphetamine (n=61) vs control (n=1297): Younger, male, and Caucasian. Methamphetamine (n=48) vs control (n=48): ↑ White matter hyperintensities, lacunes, and total burden of cSVD.
Nabaei *et al.*	2016	Twenty methamphetamine users and 21 controls (20 - 40 years old) with the median lifetime use of methamphetamine among users was 1460 grams (interquartile range from 913 to 2920 grams), with an average usage duration of 60 months.	Methamphetamine vs control ↓ Nitroglycerine-mediated dilatation markers (endothelium-independent vasodilation), NS: Flow-mediated dilatation (endothelium-dependent vasodilation), common carotid artery intima-media thickness (a marker of early atherogenesis)

↑ elevated/higher ↓ reduced/lower + positive association - negative association 7-NI: 7-nitroindazole; AKI: acute kidney injury; Akt: protein kinase B;ALP: alkaline phosphatase; ALT: alanine transaminase; AST: aspartate transaminase; ATF4: activating transcription factor 4; ATN: acute tubular necrosis; BA: bile acid; Bax: B-cell lymphoma protein 2-associated X; BBB: blood-brain barrier; Bcl-2: B-cell lymphoma protein 2; BDNF: brain-derived neurotrophic factor; BP: blood pressure; CA: d4-cholic acid; cAMP: cyclic adenosine monophosphate; CDCA: chenodeoxycholic acid; CKD: chronic kidney disease; CK-MB: creatine kinase-MB; CMECs; cardiac microvascular endothelial cells; CO: cardiac output; Cr: creatine; CRA: central retinal artery; CSE: cystathionine γ-lyase; cSVD: cerebral small vessel disease; cTnI: cardiac troponin I; CYP: cytochrome P450; CVD: cardiovascular disease; DAB: diaminobenzidine; DA: dopamine; DAT: dopamine transporter; DCA: deoxycholic acid; DHE: dihydroethidium; DLPFC: dorsolateral prefrontal cortex; D-NAME: N^G^-nitro-D-arginine-methyl ester; DOPAC: 3,4-dihydroxyphenylacetic acid; dp/dt: pressure change; DR: dopamine receptor; EB-Alb: Evans Blue-albumin; ECG: electrocardiogram; EF: ejection fraction; EDV: end-diastolic volume; eNOS: endothelial nitric oxide synthase; ERK: extracellular signal-regulated kinase 1/2; ET-1: endothelin-1; Fis1: mitochondrial fission 1 protein; FS: fractional shortening; FSGS: Focal segmental glomerulosclerosis; GABA: γ-aminobutyric acid; GCL: ganglion cell layer; GFAP: glial fibrillary acidic protein; GLUT-1: glucose transporter protein-1; Glx: glutamine + glutamate; GN: glomerulonephritis; GPx: glutathione peroxidase; GSH: glutathione; GPC: glycerophosphorylcholine; GPx: glutathione peroxidase; HAM-A: Hamilton anxiety rating scale; HAM-D: Hamilton depression rating scale; HBECs: human brain endothelial cells; HBMECs: human brain microvascular endothelial cells; HC: healthy control; HCA: hyocholic acid; H&E: hematoxylin and eosin; HDL: high-density lipoprotein; HIF-1α: hypoxia inducible factor 1-alpha; HO-1: heme oxygenase-1; HR: heart rate; HRa: hazard ratio; H_2_S: hydrogen sulfide; HUVECs: Human umbilical vein endothelial cells; HVA: homovanillic acid; ICAM: intercellular cell adhesion molecule; IgAN: Immunoglobulin A nephritis; IL: interleukin; INF: interferon; INL: inner nuclear layer; Ins: inositol; IP: intraperitoneal; IPL: inner plexiform layer; IPSC: inhibitory postsynaptic currents; ISON: isosorbide dinitrate; IV: intravenous; LCA: lithocholic acid; LDH: lactate dehydrogenase; LDL: low-density lipoprotein; LGE: late gadolinium enhancement; L-NAME: N^G^ -nitro-L-arginine methyl ester; LV: left ventricle; LVID: left ventricular internal diameter; MCP: monocyte chemoattractant protein; MDA: malondialdehyde; MDC: macrophage-derived chemokine; MIP: macrophage inflammatory protein; MMP: matrix metalloproteinases; MRS: magnetic resonance spectroscopy; MP: microparticles; MPO: myeloperoxidase; MRW: Bruch's membrane opening minimum rim width; mTOR: mammalian target of rapamycin; NAA: *N*-acetyl aspartate; NAAG: *N*-acetylaspartylglutamate; NAc: nucleus accumbens; NACA: *N*-acetylcysteine amide; NF-κB: nuclear factor kappa B; NO: nitrogen oxide; NOARG: N^G^ -nitro-L-arginine; NOS: nitric oxide synthase; NOX: NADPH oxidase; Nrf2: nuclear factor erythroid 2-related factor; NS: not significant; ONL: outer nuclear layer; PCh: phosphorylcholine; PCr: phosphocreatine; PECAM-1: platelet endothelial cell adhesion molecule-1; PK: penetrating keratoplasty; PI3K: phosphatidylinositol-3-kinase; RFNL: retinal nerve fibre layer thickness; ROS: reactive oxygen species; RPE: retinal pigmen epithelium; RV: right ventricle; SFN: sulforaphane; SIRT: sirtuin; SNP: sodium nitroprusside; SOD: superoxide dismutase; SPF: specific-pathogen-free; SV: stroke volume; TARC: thymus- and activation-regulated chemokine; TG: triglycerides; TLCA: taurolithocholic acid: TLR: Toll-like receptor; TNF-α: tumour necrosis factor alpha; TR: tricuspid regurgitation; TRP: transient receptor potential; TUNEL: terminal deoxynucleotidyl transferase deoxyuridine triphosphate nick end labelling; UDCA: ursodeoxycholic acid; VCAM: vascular cell adhesion molecule; VE: vascular endothelial; VEGF: vascular endothelial growth factor; WT: wild-type; ZO-1: zonula occludens-1.

## References

[1] Jones CM, Compton WM, Mustaquim D (2020). Patterns and Characteristics of Methamphetamine Use Among Adults - United States, 2015-2018. MMWR Morb Mortal Wkly Rep.

[2] UNODC (2023). World Drug Report 2023. United Nations.

[3] Khoshnevis S, Smolensky MH, Haghayegh S, Castriotta RJ, Hermida RC, Diller KR (2023). Recommended timing of medications that impact sleep and wakefulness: A review of the American Prescribers' Digital Reference. Sleep Med Rev.

[4] Moszczynska A (2021). Current and Emerging Treatments for Methamphetamine Use Disorder. Curr Neuropharmacol.

[5] Alam-mehrjerdi Z, Mokri A, Dolan K (2015). Methamphetamine use and treatment in Iran: A systematic review from the most populated Persian Gulf country. Asian J Psychiatr.

[6] Ramli FF, Shuid AN, Pakri Mohamed RM, Tg Abu Bakar Sidik TMI, Naina Mohamed I (2019). Health-Seeking Behavior for Erectile Dysfunction in Methadone Maintenance Treatment Patients. Int J Environ Res Public Health.

[7] Ramli FF, Tg Abu Bakar Sidik TMI, Naina Mohamed I (2020). Sexual Inactivity in Methadone Maintenance Treatment Patients. Int J Environ Res Public Health.

[8] Everett NA, Baracz SJ, Cornish JL (2020). The effect of chronic oxytocin treatment during abstinence from methamphetamine self-administration on incubation of craving, reinstatement, and anxiety. Neuropsychopharmacology.

[9] Greening DW, Notaras M, Chen M, Xu R, Smith JD, Cheng L (2021). Chronic methamphetamine interacts with BDNF Val66Met to remodel psychosis pathways in the mesocorticolimbic proteome. Mol Psychiatry.

[10] Curran L, Nah G, Marcus GM, Tseng Z, Crawford MH, Parikh NI (2022). Clinical Correlates and Outcomes of Methamphetamine-Associated Cardiovascular Diseases in Hospitalized Patients in California. J Am Heart Assoc.

[11] Abbruscato TJ, Trippier PC (2018). DARK Classics in Chemical Neuroscience: Methamphetamine. ACS Chem Neurosci.

[12] Pauly RC, Bhimani RV, Li JX, Blough BE, Landavazo A, Park J (2023). Distinct Effects of Methamphetamine Isomers on Limbic Norepinephrine and Dopamine Transmission in the Rat Brain. ACS Chem Neurosci.

[13] Kunalan V, Nic Daéid N, Kerr WJ, Buchanan HA, McPherson AR (2009). Characterization of route specific impurities found in methamphetamine synthesized by the Leuckart and reductive amination methods. Anal Chem.

[14] Vearrier D, Greenberg MI, Miller SN, Okaneku JT, Haggerty DA (2012). Methamphetamine: history, pathophysiology, adverse health effects, current trends, and hazards associated with the clandestine manufacture of methamphetamine. Dis Mon.

[15] Schepers RJ, Oyler JM, Joseph RE Jr, Cone EJ, Moolchan ET, Huestis MA (2003). Methamphetamine and amphetamine pharmacokinetics in oral fluid and plasma after controlled oral methamphetamine administration to human volunteers. Clin Chem.

[16] Wang T, Shen B, Wu H, Hu J, Xu H, Shen M (2018). Disappearance of R/S-methamphetamine and R/S-amphetamine from human scalp hair after discontinuation of methamphetamine abuse. Forensic Sci Int.

[17] Chomchai C, Chomchai S, Kitsommart R (2016). Transfer of Methamphetamine (MA) into Breast Milk and Urine of Postpartum Women who Smoked MA Tablets during Pregnancy: Implications for Initiation of Breastfeeding. J Hum Lact.

[18] Bartu A, Dusci LJ, Ilett KF (2009). Transfer of methylamphetamine and amphetamine into breast milk following recreational use of methylamphetamine. Br J Clin Pharmacol.

[19] Volkow ND, Fowler JS, Wang GJ, Shumay E, Telang F, Thanos PK (2010). Distribution and pharmacokinetics of methamphetamine in the human body: clinical implications. PLoS One.

[20] Harris DS, Boxenbaum H, Everhart ET, Sequeira G, Mendelson JE, Jones RT (2003). The bioavailability of intranasal and smoked methamphetamine. Clin Pharmacol Ther.

[21] de la Torre R, Yubero-Lahoz S, Pardo-Lozano R, Farré M (2012). MDMA, methamphetamine, and CYP2D6 pharmacogenetics: what is clinically relevant?. Front Genet.

[22] Shima N, Tsutsumi H, Kamata T, Nishikawa M, Katagi M, Miki A (2006). Direct determination of glucuronide and sulfate of p-hydroxymethamphetamine in methamphetamine users' urine. J Chromatogr B Analyt Technol Biomed Life Sci.

[23] Ramamoorthy Y, Tyndale RF, Sellers EM (2001). Cytochrome P450 2D6.1 and cytochrome P450 2D6.10 differ in catalytic activity for multiple substrates. Pharmacogenetics.

[24] Matsusue A, Ikeda T, Tani N, Waters B, Hara K, Kashiwagi M (2018). Association between cytochrome P450 2D6 polymorphisms and body fluid methamphetamine concentrations in Japanese forensic autopsy cases. Forensic Sci Int.

[25] Wagner DJ, Sager JE, Duan H, Isoherranen N, Wang J (2017). Interaction and Transport of Methamphetamine and its Primary Metabolites by Organic Cation and Multidrug and Toxin Extrusion Transporters. Drug Metab Dispos.

[26] Strang J, Bearn J, Farrell M, Finch E, Gossop M, Griffiths P (1998). Route of drug use and its implications for drug effect, risk of dependence and health consequences. Drug Alcohol Rev.

[27] Hayley AC, Shiferaw B, Rositano J, Downey LA (2023). Acute neurocognitive and subjective effects of oral methamphetamine with low doses of alcohol: A randomised controlled trial. J Psychopharmacol.

[28] Sulzer D, Rayport S (1990). Amphetamine and other psychostimulants reduce pH gradients in midbrain dopaminergic neurons and chromaffin granules: a mechanism of action. Neuron.

[29] Bisagno V, Cadet JL (2022). Methamphetamine and MDMA Neurotoxicity: Biochemical and Molecular Mechanisms. In: Kostrzewa RM, editor. Handbook of Neurotoxicity. New York, NY: Springer.

[30] Branch SY, Beckstead MJ (2012). Methamphetamine produces bidirectional, concentration-dependent effects on dopamine neuron excitability and dopamine-mediated synaptic currents. J Neurophysiol.

[31] Moreira da Silva Santos A, Kelly JP, Dockery P, Doyle KM (2019). Effect of a binge-like dosing regimen of methamphetamine on dopamine levels and tyrosine hydroxylase expressing neurons in the rat brain. Prog Neuropsychopharmacol Biol Psychiatry.

[32] Jayanthi S, Daiwile AP, Cadet JL (2021). Neurotoxicity of methamphetamine: Main effects and mechanisms. Exp Neurol.

[33] Su H, Wang X, Bai J, Fan Y, Du Y, Wei Z (2022). Role of dopamine D3 receptors in methamphetamine-induced behavioural sensitization and the characterization of dopamine receptors (D1R-D5R) gene expression in the brain. Folia Neuropathol.

[34] Baladi MG, Newman AH, Nielsen SM, Hanson GR, Fleckenstein AE (2014). Dopamine D(3) receptors contribute to methamphetamine-induced alterations in dopaminergic neuronal function: role of hyperthermia. Eur J Pharmacol.

[35] Gibson AS, West PJ, Keefe KA (2022). Effects of methamphetamine-induced neurotoxicity on striatal long-term potentiation. Psychopharmacology (Berl).

[36] Kupchik YM, Kalivas PW (2017). The Direct and Indirect Pathways of the Nucleus Accumbens are not What You Think. Neuropsychopharmacology.

[37] Miyamoto Y, Iida A, Sato K, Muramatsu S, Nitta A (2014). Knockdown of dopamine D₂ receptors in the nucleus accumbens core suppresses methamphetamine-induced behaviors and signal transduction in mice. Int J Neuropsychopharmacol.

[38] Di Monte DA, Royland JE, Jakowec MW, Langston JW (1996). Role of nitric oxide in methamphetamine neurotoxicity: protection by 7-nitroindazole, an inhibitor of neuronal nitric oxide synthase. J Neurochem.

[39] Bowyer JF, Clausing P, Gough B, Slikker W Jr, Holson RR (1995). Nitric oxide regulation of methamphetamine-induced dopamine release in caudate/putamen. Brain Res.

[40] Su H, Chen T, Zhong N, Jiang H, Du J, Xiao K (2020). Decreased GABA concentrations in left prefrontal cortex of methamphetamine dependent patients: A proton magnetic resonance spectroscopy study. J Clin Neurosci.

[41] Watling SE, Jagasar S, McCluskey T, Warsh J, Rhind SG, Truong P (2022). Imaging oxidative stress in brains of chronic methamphetamine users: A combined 1H-magnetic resonance spectroscopy and peripheral blood biomarker study. Front Psychiatry.

[42] Batra V, Murnane KS, Knox B, Edinoff AN, Ghaffar Y, Nussdorf L (2022). Early onset cardiovascular disease related to methamphetamine use is most striking in individuals under 30: A retrospective chart review. Addict Behav Rep.

[43] Jariwal R, Narang V, Raza N, Mann B, Bhandohal J, Valdez M (2021). Echocardiographic Findings in Heart Failure Patients With Methamphetamine Use: A Case-Control Study. Cureus.

[44] Bhatia HS, Nishimura M, Dickson S, Adler E, Greenberg B, Thomas IC (2021). Clinical and echocardiographic outcomes in heart failure associated with methamphetamine use and cessation. Heart.

[45] Zheng XZ, Shi YY, Chen KQ, Qiao XL, Wang LY (2019). Evaluation of regional myocardial perfusion in methamphetamine abusers using real-time myocardial contrast echocardiography. Med Ultrason.

[46] Yu H, Peng Y, Dong W, Shen B, Yang G, Nie Q (2023). Nrf2 attenuates methamphetamine-induced myocardial injury by regulating oxidative stress and apoptosis in mice. Hum Exp Toxicol.

[47] Kolluru GK, Glawe JD, Pardue S, Kasabali A, Alam S, Rajendran S (2022). Methamphetamine causes cardiovascular dysfunction via cystathionine gamma lyase and hydrogen sulfide depletion. Redox Biol.

[48] Abdullah CS, Aishwarya R, Alam S, Morshed M, Remex NS, Nitu S (2020). Methamphetamine induces cardiomyopathy by Sigmar1 inhibition-dependent impairment of mitochondrial dynamics and function. Commun Biol.

[49] Xiao Q, Ying J, Xiang L, Zhang C (2018). The biologic effect of hydrogen sulfide and its function in various diseases. Medicine (Baltimore).

[50] Marcinko MC, Darrow AL, Tuia AJ, Shohet RV (2019). Sex influences susceptibility to methamphetamine cardiomyopathy in mice. Physiol Rep.

[51] Zhang J, Nguyen AH, Jilani D, Trigo Torres RS, Schmiess-Heine L, Le T (2023). Consecutive treatments of methamphetamine promote the development of cardiac pathological symptoms in zebrafish. PLoS One.

[52] Stokes MB, Thoi F, Scherer DJ, Win KTH, Kaye DM, Teo KS (2022). Cardiovascular magnetic resonance imaging characteristics in patients with methamphetamine-associated cardiomyopathy. J Cardiovasc Magn Reson.

[53] Akhgari M, Mobaraki H, Etemadi-Aleagha A (2017). Histopathological study of cardiac lesions in methamphetamine poisoning-related deaths. Daru.

[54] Freeling JL, McFadden LM (2020). The emergence of cardiac changes following the self-administration of methamphetamine. Drug Alcohol Depend.

[55] Chavva H, Brazeau DA, Denvir J, Primerano DA, Fan J, Seeley SL (2021). Methamphetamine-induced changes in myocardial gene transcription are sex-dependent. BMC Genomics.

[56] Rorabaugh BR, Seeley SL, Stoops TS, D'Souza MS (2017). Repeated exposure to methamphetamine induces sex-dependent hypersensitivity to ischemic injury in the adult rat heart. PLoS One.

[57] Chavva H, Rorabaugh BR (2022). Methamphetamine Use During the First or Second Half of Pregnancy Worsens Cardiac Ischemic Injury in Adult Female Offspring. Physiol Res.

[58] Vaupel DB, Schindler CW, Chefer S, Belcher AM, Ahmet I, Scheidweiler KB (2016). Delayed emergence of methamphetamine's enhanced cardiovascular effects in nonhuman primates during protracted methamphetamine abstinence. Drug Alcohol Depend.

[59] Dague A, Chavva H, Brazeau DA, Denvir J, Rorabaugh BR (2022). Maternal use of methamphetamine induces sex-dependent changes in myocardial gene expression in adult offspring. Physiol Rep.

[60] Zhao T, Zhai C, Song H, Wu Y, Ge C, Zhang Y (2020). Methamphetamine-Induced Cognitive Deficits and Psychiatric Symptoms Are Associated with Serum Markers of Liver Damage. Neurotox Res.

[61] Chen L-J, Li X-W, Liu Y, Liu J-L, Yang J-Z, Li J-H (2023). Propionate, rather than acetate or butyrate, ameliorates methamphetamine-induced hepatotoxicity and enterotoxicity in mice by downregulating the TLR4/NF-κB pathway. Journal of Functional Foods.

[62] Halpin LE, Yamamoto BK (2012). Peripheral ammonia as a mediator of methamphetamine neurotoxicity. J Neurosci.

[63] Koriem KM, Soliman RE (2014). Chlorogenic and caftaric acids in liver toxicity and oxidative stress induced by methamphetamine. J Toxicol.

[64] Wang LB, Chen LJ, Wang Q, Xie XL (2022). Silencing the Tlr4 Gene Alleviates Methamphetamine-Induced Hepatotoxicity by Inhibiting Lipopolysaccharide-Mediated Inflammation in Mice. Int J Mol Sci.

[65] Wang Q, Wei LW, Xiao HQ, Xue Y, Du SH, Liu YG (2017). Methamphetamine induces hepatotoxicity via inhibiting cell division, arresting cell cycle and activating apoptosis: In vivo and in vitro studies. Food Chem Toxicol.

[66] Zhang KK, Liu JL, Chen LJ, Li JH, Yang JZ, Xu LL (2022). Gut microbiota mediates methamphetamine-induced hepatic inflammation via the impairment of bile acid homeostasis. Food Chem Toxicol.

[67] Ma Y, Wu H, Wang H, Chen F, Xie Z, Zhang Z (2021). Psychiatric Comorbidities and Liver Injury Are Associated With Unbalanced Plasma Bile Acid Profile During Methamphetamine Withdrawal. Front Endocrinol (Lausanne).

[68] Chen LJ, He JT, Pan M, Liu JL, Zhang KK, Li JH (2021). Antibiotics Attenuate Methamphetamine-Induced Hepatotoxicity by Regulating Oxidative Stress and TLR4/MyD88/Traf6 Axis. Front Pharmacol.

[69] Xie XL, Zhou WT, Zhang KK, Chen LJ, Wang Q (2018). METH-Induced Neurotoxicity Is Alleviated by Lactulose Pretreatment Through Suppressing Oxidative Stress and Neuroinflammation in Rat Striatum. Front Neurosci.

[70] Azizi S, Kheirandish R, Dabiri S, Lakzaee M (2023). Adverse effects of methamphetamine on vital organs of male rats: Histopathological and immunohistochemical investigations. Iran J Basic Med Sci.

[71] Carvalho M, Milhazes N, Remião F, Borges F, Fernandes E, Amado F (2004). Hepatotoxicity of 3,4-methylenedioxyamphetamine and alpha-methyldopamine in isolated rat hepatocytes: formation of glutathione conjugates. Arch Toxicol.

[72] Willson C (2019). Sympathomimetic amine compounds and hepatotoxicity: Not all are alike-Key distinctions noted in a short review. Toxicol Rep.

[73] Karch SB, Stephens BG, Ho CH (1999). Methamphetamine-related deaths in San Francisco: demographic, pathologic, and toxicologic profiles. J Forensic Sci.

[74] Merchant K, Schammel C, Fulcher J (2019). Acute Methamphetamine-Induced Hepatic and Pancreatic Ischemia. Am J Forensic Med Pathol.

[75] Filipović B, Marković O, Đurić V, Filipović B (2018). Cognitive Changes and Brain Volume Reduction in Patients with Nonalcoholic Fatty Liver Disease. Can J Gastroenterol Hepatol.

[76] Seo SW, Gottesman RF, Clark JM, Hernaez R, Chang Y, Kim C (2016). Nonalcoholic fatty liver disease is associated with cognitive function in adults. Neurology.

[77] Huang Y, Chundury RV, Timperley BD, Terp PA, Krueger RR, Yeh S (2022). Ophthalmic complications associated with methamphetamine use disorder. Am J Ophthalmol Case Rep.

[78] Mahjoob M, Heydarian S (2022). Long-term effects of methamphetamine abuse on visual evoked potentials. Ophthalmic Physiol Opt.

[79] Wijaya J, Salu P, Leblanc A, Bervoets S (1999). Acute unilateral visual loss due to a single intranasal methamphetamine abuse. Bull Soc Belge Ophtalmol.

[80] Wallace RT, Brown GC, Benson W, Sivalingham A (1992). Sudden retinal manifestations of intranasal cocaine and methamphetamine abuse. Am J Ophthalmol.

[81] Shaw HE Jr, Lawson JG, Stulting RD (1985). Amaurosis fugax and retinal vasculitis associated with methamphetamine inhalation. J Clin Neuroophthalmol.

[82] Franco J, Bennett A, Patel P, Waldrop W, McCulley J (2022). Methamphetamine-Induced Keratitis Case Series. Cornea.

[83] Poulsen EJ, Mannis MJ, Chang SD (1996). Keratitis in methamphetamine abusers. Cornea.

[84] Chuck RS, Williams JM, Goldberg MA, Lubniewski AJ (1996). Recurrent corneal ulcerations associated with smokeable methamphetamine abuse. Am J Ophthalmol.

[85] Wu H, Hu Y, Shi XR, Xu F, Jiang CY, Huang R (2016). Keratopathy due to ophthalmic drug abuse with corneal melting and perforation presenting as Mooren-like ulcer: A case report. Exp Ther Med.

[86] Lee M, Leskova W, Eshaq RS, Harris NR (2020). Acute changes in the retina and central retinal artery with methamphetamine. Exp Eye Res.

[87] Guo J, Tang W, Liu W, Zhang Y, Wang L, Wang W (2019). Bilateral methamphetamine-induced ischemic retinopathy. Am J Ophthalmol Case Rep.

[88] Tang YF, Chong E (2022). Vision loss and methamphetamine use. Med J Aust.

[89] Lee M, Leskova W, Eshaq RS, Harris NR (2021). Retinal hypoxia and angiogenesis with methamphetamine. Exp Eye Res.

[90] Melo P, Zanon-Moreno V, Alves CJ, Magalhães A, Tavares MA, Pinazo-Duran MD (2010). Oxidative stress response in the adult rat retina and plasma after repeated administration of methamphetamine. Neurochem Int.

[91] Turan Ç, Şenormancı G, Neşelioğlu S, Budak Y, Erel Ö, Şenormancı Ö (2023). Oxidative Stress and Inflammatory Biomarkers in People with Methamphetamine Use Disorder. Clin Psychopharmacol Neurosci.

[92] Talebnejad MR, Khazaei P, Jahanbani-Ardakani H, Saberikia Z, Moghimi Sarani E, Khalili MR (2020). Effects of chronic methamphetamine abuse on the retinal nerve fiber layer, ganglion cell layer and Bruch's membrane opening minimum rim width. Neurotoxicology.

[93] Lai H, Zeng H, Zhang C, Wang L, Tso MO, Lai S (2009). Toxic effect of methamphetamine on the retina of CD1 mice. Curr Eye Res.

[94] Kumar RL, Kaiser PK, Lee MS (2006). Crystalline retinopathy from nasal ingestion of methamphetamine. Retina.

[95] Gurel A (2016). Multisystem toxicity after methamphetamine use. Clin Case Rep.

[96] Ago M, Ago K, Hara K, Kashimura S, Ogata M (2006). Toxicological and histopathological analysis of a patient who died nine days after a single intravenous dose of methamphetamine: a case report. Leg Med (Tokyo).

[97] Jones ES, Rayner BL (2015). Hypertension, end-stage renal disease and mesangiocapillary glomerulonephritis in methamphetamine users. S Afr Med J.

[98] Baradhi KM, Pathireddy S, Bose S, Aeddula NR (2019). Methamphetamine (N-methylamphetamine)-induced renal disease: underevaluated cause of end-stage renal disease (ESRD). BMJ Case Rep.

[99] Grace Choung HY, Nast CC, Haas M, Lin M, Yamashita M, Hou J (2024). Spectrum of Kidney Biopsy Findings Associated With Methamphetamine Use. Kidney Int Rep.

[100] Chansaengpetch N, Worasuwannarak W, Worawichawong S (2023). Methamphetamine-induced profound rhabdomyolysis and myoglobin cast nephropathy: A case report and a literature review. J Forensic Leg Med.

[101] Isoardi KZ, Mudge DW, Harris K, Dimeski G, Buckley NA (2020). Methamphetamine intoxication and acute kidney injury: A prospective observational case series. Nephrology (Carlton).

[102] Ishigami A, Tokunaga I, Gotohda T, Kubo S (2003). Immunohistochemical study of myoglobin and oxidative injury-related markers in the kidney of methamphetamine abusers. Leg Med (Tokyo).

[103] Amanollahi A, Mehrabi Y, Sedighi M, Basir Ghafouri H, Zahedi A, Shadnia S (2023). Assessment of renal function indexes in methamphetamine or tramadol intoxication adults to the emergency departments: a systematic review and meta-analysis. BMC Emerg Med.

[104] Pajoum A, Fahim F, Akhlaghdoust M, Zamani N, Amirfirooz Z, Dehdehasti M (2018). Rhabdomyolysis and Acute Poisoning; a Brief Report. Emerg (Tehran).

[105] Godrati S, Pezeshgi A, Valizadeh R, Kellner SJ, Radfar SR (2020). Acute and delayed nephropathy due to methamphetamine abuse. Journal of Nephropathology.

[106] Moore KP, Holt SG, Patel RP, Svistunenko DA, Zackert W, Goodier D (1998). A causative role for redox cycling of myoglobin and its inhibition by alkalinization in the pathogenesis and treatment of rhabdomyolysis-induced renal failure. J Biol Chem.

[107] Fukunaga M, Yura T, Badr KF (1995). Stimulatory effect of 8-Epi-PGF2 alpha, an F2-isoprostane, on endothelin-1 release. J Cardiovasc Pharmacol.

[108] De Miguel C, Speed JS, Kasztan M, Gohar EY, Pollock DM (2016). Endothelin-1 and the kidney: new perspectives and recent findings. Curr Opin Nephrol Hypertens.

[109] Zhang X, Tobwala S, Ercal N (2012). N-acetylcysteine amide protects against methamphetamine-induced tissue damage in CD-1 mice. Hum Exp Toxicol.

[110] Koriem KM, Abdelhamid AZ, Younes HF (2013). Caffeic acid protects tissue antioxidants and DNA content in methamphetamine induced tissue toxicity in Sprague Dawley rats. Toxicol Mech Methods.

[111] Dong W, Wan J, Yu H, Shen B, Yang G, Nie Q (2023). Nrf2 protects against methamphetamine-induced nephrotoxicity by mitigating oxidative stress and autophagy in mice. Toxicol Lett.

[112] Najafian B, Fogo AB, Lusco MA, Alpers CE (2017). AJKD Atlas of Renal Pathology: Myoglobin Cast Nephropathy. Am J Kidney Dis.

[113] Alberts B, Johnson A, Lewis J, Raff M, Roberts K, Walter P (2002). Blood vessels and endothelial cells. Molecular Biology of the Cell 4th edition: Garland Science.

[114] Félétou M (2011). Integrated Systems Physiology: from Molecule to Function to Disease. The Endothelium: Part 1: Multiple Functions of the Endothelial Cells—Focus on Endothelium-Derived Vasoactive Mediators. San Rafael (CA): Morgan & Claypool Life Sciences.

[115] Bautch VL, Caron KM (2015). Blood and lymphatic vessel formation. Cold Spring Harb Perspect Biol.

[116] Sweeney MD, Sagare AP, Zlokovic BV (2018). Blood-brain barrier breakdown in Alzheimer disease and other neurodegenerative disorders. Nature Reviews Neurology.

[117] Ramirez SH, Potula R, Fan S, Eidem T, Papugani A, Reichenbach N (2009). Methamphetamine disrupts blood-brain barrier function by induction of oxidative stress in brain endothelial cells. J Cereb Blood Flow Metab.

[118] Hwang JS, Cha EH, Park B, Ha E, Seo JH (2020). PBN inhibits a detrimental effect of methamphetamine on brain endothelial cells by alleviating the generation of reactive oxygen species. Arch Pharm Res.

[119] Abdul Muneer PM, Alikunju S, Szlachetka AM, Murrin LC, Haorah J (2011). Impairment of brain endothelial glucose transporter by methamphetamine causes blood-brain barrier dysfunction. Mol Neurodegener.

[120] Nazari A, Zahmatkesh M, Mortaz E, Hosseinzadeh S (2018). Effect of methamphetamine exposure on the plasma levels of endothelial-derived microparticles. Drug Alcohol Depend.

[121] Martins T, Burgoyne T, Kenny BA, Hudson N, Futter CE, Ambrósio AF (2013). Methamphetamine-induced nitric oxide promotes vesicular transport in blood-brain barrier endothelial cells. Neuropharmacology.

[122] Chang JH, Greene C, Frudd K, Araujo Dos Santos L, Futter C, Nichols BJ (2022). Methamphetamine enhances caveolar transport of therapeutic agents across the rodent blood-brain barrier. Cell Rep Med.

[123] Seo JW, Jones SM, Hostetter TA, Iliff JJ, West GA (2016). Methamphetamine induces the release of endothelin. J Neurosci Res.

[124] Nabaei G, Oveisgharan S, Ghorbani A, Fatehi F (2016). Impaired arterial smooth muscle cell vasodilatory function in methamphetamine users. J Neurol Sci.

[125] Zhu Z, Vanderschelden B, Lee SJ, Blackwill H, Shafie M, Soun JE (2023). Methamphetamine use increases the risk of cerebral small vessel disease in young patients with acute ischemic stroke. Sci Rep.

[126] Ma J, Wan J, Meng J, Banerjee S, Ramakrishnan S, Roy S (2014). Methamphetamine induces autophagy as a pro-survival response against apoptotic endothelial cell death through the Kappa opioid receptor. Cell Death Dis.

[127] Jumnongprakhon P, Govitrapong P, Tocharus C, Tocharus J (2016). Inhibitory effect of melatonin on cerebral endothelial cells dysfunction induced by methamphetamine via NADPH oxidase-2. Brain Res.

[128] Cai D, Huang E, Luo B, Yang Y, Zhang F, Liu C (2016). Nupr1/Chop signal axis is involved in mitochondrion-related endothelial cell apoptosis induced by methamphetamine. Cell Death Dis.

[129] Chen R, Huang P, Wei S, Zhang C, Lai X, Wang H (2022). Methamphetamine exposure increases cardiac microvascular permeability by activating the VEGF-PI3K-Akt-eNOS signaling pathway, reversed by Bevacizumab. Hum Exp Toxicol.

